# Quantitative high-throughput screening assays for the discovery and development of SIRPα-CD47 interaction inhibitors

**DOI:** 10.1371/journal.pone.0218897

**Published:** 2019-07-05

**Authors:** Thomas W. Miller, Joshua D. Amason, Elsa D. Garcin, Laurence Lamy, Patricia K. Dranchak, Ryan Macarthur, John Braisted, Jeffrey S. Rubin, Teresa L. Burgess, Catherine L. Farrell, David D. Roberts, James Inglese

**Affiliations:** 1 Paradigm Shift Therapeutics LLC, Rockville, Maryland, United States of America; 2 Laboratory of Pathology, Center for Cancer Research, National Cancer Institute, National Institutes of Health, Bethesda, Maryland, United States of America; 3 Department of Chemistry and Biochemistry, University of Maryland Baltimore County, Catonsville, Maryland, United States of America; 4 Division of Preclinical Innovation, National Center for Advancing Translational Sciences, National Institutes of Health, Rockville, Maryland, United States of America; University of Pittsburgh School of Medicine, UNITED STATES

## Abstract

CD47 is an immune checkpoint molecule that downregulates key aspects of both the innate and adaptive anti-tumor immune response via its counter receptor SIRPα, and it is expressed at high levels in a wide variety of tumor types. This has led to the development of biologics that inhibit SIRPα engagement including humanized CD47 antibodies and a soluble SIRPα decoy receptor that are currently undergoing clinical trials. Unfortunately, toxicological issues, including anemia related to on-target mechanisms, are barriers to their clinical advancement. Another potential issue with large biologics that bind CD47 is perturbation of CD47 signaling through its high-affinity interaction with the matricellular protein thrombospondin-1 (TSP1). One approach to avoid these shortcomings is to identify and develop small molecule molecular probes and pretherapeutic agents that would (1) selectively target SIRPα or TSP1 interactions with CD47, (2) provide a route to optimize pharmacokinetics, reduce on-target toxicity and maximize tissue penetration, and (3) allow more flexible routes of administration. As the first step toward this goal, we report the development of an automated quantitative high-throughput screening (qHTS) assay platform capable of screening large diverse drug-like chemical libraries to discover novel small molecules that inhibit CD47-SIRPα interaction. Using time-resolved Förster resonance energy transfer (TR-FRET) and bead-based luminescent oxygen channeling assay formats (AlphaScreen), we developed biochemical assays, optimized their performance, and individually tested them in small-molecule library screening. Based on performance and low false positive rate, the LANCE TR-FRET assay was employed in a ~90,000 compound library qHTS, while the AlphaScreen oxygen channeling assay served as a cross-validation orthogonal assay for follow-up characterization. With this multi-assay strategy, we successfully eliminated compounds that interfered with the assays and identified five compounds that inhibit the CD47-SIRPα interaction; these compounds will be further characterized and later disclosed. Importantly, our results validate the large library qHTS for antagonists of CD47-SIRPα interaction and suggest broad applicability of this approach to screen chemical libraries for other protein-protein interaction modulators.

## Introduction

The immune system can detect and eliminate cancer cells [1–4]. However, tumors arise and persist largely due to acquired adaptations that enable tumor cells to avoid immune destruction by restricting innate immune surveillance [5], limiting cytotoxic T cell activity [6], and promoting overall immune tolerance [7]. Successful new immuno-oncology drugs such as antibodies targeting the immune checkpoints PD-1/PD-L1 and CTLA-4(CD80/CD86) remove tumor barriers to the adaptive immune response, enabling T cells to attack tumors, and lead to remarkably durable responses in some melanoma [8,9], lung [10], head and neck [11], and bladder [12] cancer patients. Other immunotherapies that modulate this adaptive immune response, including chimeric antigen receptor (CAR) T cells [13] and dendritic cell vaccines [14], also show promise. However, overall clinical response rates remain low for T cell therapies in solid tumors [15,16], highlighting the need for additional immunotherapeutic strategies.

CD47 is expressed on the surface of normal cells and binds to its counter-receptor SIRPα expressed on macrophages to inhibit phagocytosis [17–20]. In healthy tissues, this forms the basis of innate immune tolerance (the “Don’t Eat Me” signal). Decreases in CD47 expression promote the removal of aged or damaged cells [17], while increased expression facilitates the preservation of nascent hematopoietic stem cells [21]. As little as ~2-fold changes in CD47 expression can affect its regulation of cell turnover [21–24].

Some types of tumor cells have co-opted the CD47-SIRPα signaling axis by elevating their CD47 expression [21,25–38] to downregulate phagocytosis and avoid innate immune surveillance. Secondarily, decreased phagocytosis impedes anti-tumor adaptive immunity by inhibiting antigen presentation and anti-tumor T cell priming by SIRPα-expressing dendritic cells, microglial cells, and tumor-associated macrophages [39–44]. A growing number of hematological and solid tumors are known to express elevated CD47 levels, which correlate with poorer prognosis [29,45,46]. CD47-targeting therapies have extensive preclinical validation, and targeting CD47 with monoclonal antibodies (MAbs) in xenograft models of ovarian, colon, breast, bladder, lung, pancreatic carcinoma, glioma, and leiomyosarcoma enhances macrophage activity and tumor elimination [29,45,46]. Using syngeneic models, we and others demonstrated that the anti-tumor efficacy of targeting CD47 requires both innate and adaptive immune activation for breast and colorectal cancer, melanoma, and B cell lymphomas [41,43,47,48]. We showed that in preclinical models of metastatic breast cancer, disrupting CD47 with MAbs or antisense suppression increased chemotherapeutic and trastuzumab/Herceptin-mediated clearance of tumors, and also prevented lung metastases and protected the heart from cardiomyopathic effects of DNA-damaging chemotherapy [30,49]. Therefore, a CD47-targeting therapeutic has strong potential for treatment of a broad range of tumor types through multiple immune- and non-immune-mediated mechanisms.

Humanized MAbs and a SIRPα-Fc decoy receptor targeting CD47 are now in early stages of clinical development based on the compelling body of preclinical results and human tumor expression data. Recently, a combination of rituximab and an anti-CD47 antibody (Hu5F9) successfully completed a phase 1b safety study and showed anecdotal evidence of response in relapsed or refractory non-Hodgkin’s lymphoma [50]. However, biologics targeting CD47 in clinical trials have exhibited clinical liabilities including 1) on-target toxicities leading to hemolytic anemia, neutropenia, and thrombocytopenia [50–54], 2) limited/variable tissue penetration and distribution into tumors, and 3) a large CD47 antigen sink in the vascular compartment [55].

**Fig 1:**
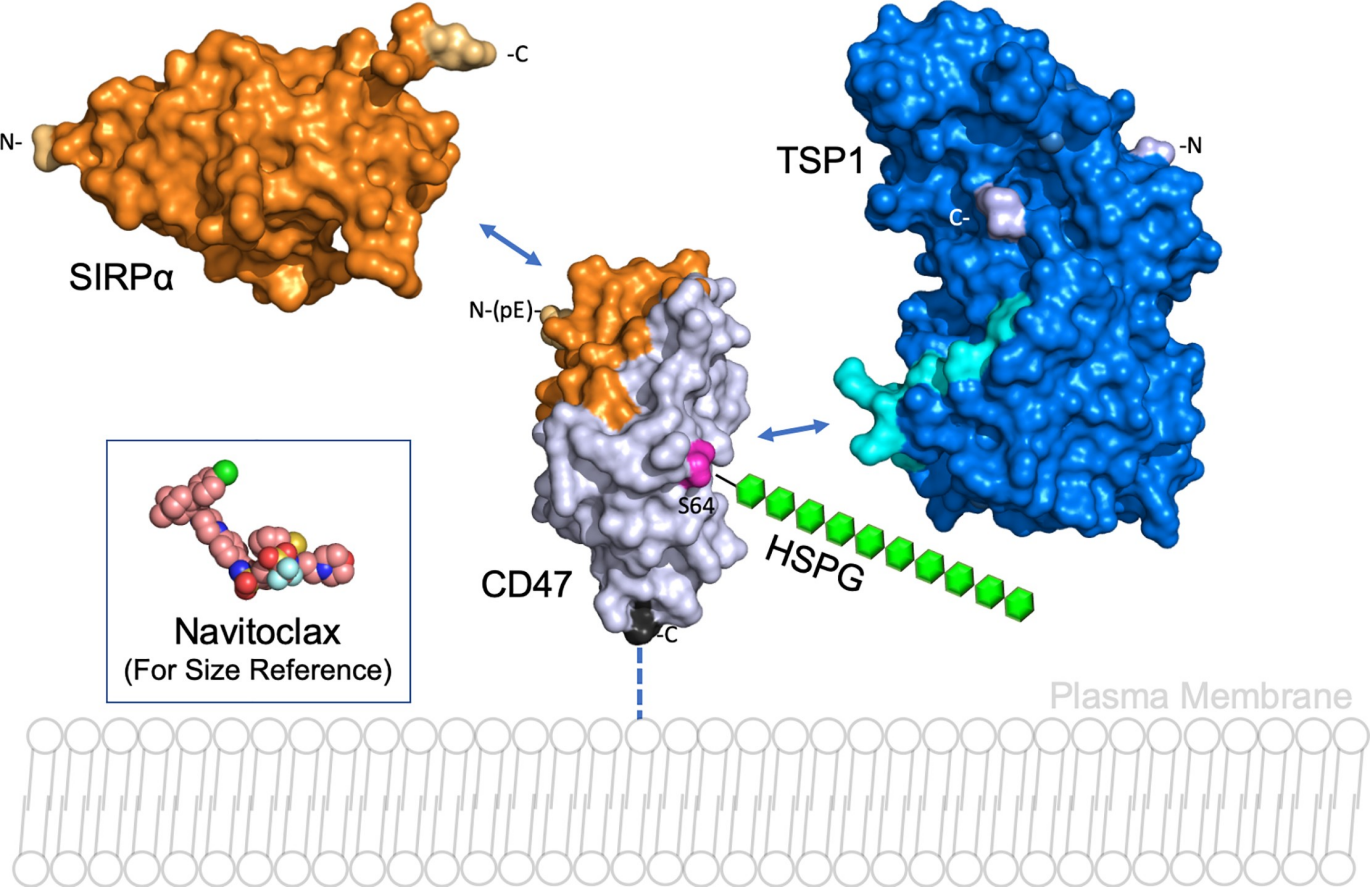
SIRPα and TSP1 interaction with CD47. The SIRPα-CD47 interface is defined by the co-crystal structure (PDB 2JJT) and is represented on the extracellular domain surfaces of CD47 (residues 20–136; gray) and SIRPα (residues 31–149; orange). The TSP1 interaction with CD47 is not well-defined but depends on a heparan-sulfate proteoglycan modification (HSPG; green) at CD47 Ser64 (magenta) and can be functionally minimized to the TSP1 peptide known as 7N3 (_1120_FIRVVMYEGKK_1130_, light blue) shown on the surface of the C-terminal CD47-interacting domain of TSP1 (E3CaG1; PDB 1UX6; residues 834–1170; blue). As a molecular size reference, the clinical stage protein-protein interaction (PPI) inhibitor navitoclax (974.6 Da) is shown (PDB 4LVT). PPI inhibitors are generally larger small molecules, and navitoclax is roughly 2x the mass of a typical small molecule drug. The N- and C-termini of the constructs are indicated.

Small molecules offer unique advantages to maximize the therapeutic benefit of disrupting the CD47-SIRPα interaction. These include the potential to 1) optimize the half-life to reduce side effects due to a long period of high target occupancy, allowing for better management of efficacy vs. toxicity; 2) increase tissue and solid tumor distribution; 3) develop oral bioavailability to increase patient convenience, compliance and reduce the cost of treatment. Additionally, the tumor-immune biology of CD47 is complex and involves its other ligand thrombospondin-1 (TSP1) [56–58]. We and others have shown that selectively blocking TSP1-CD47 interaction leads to protection of T cells from anti-tumor radiation therapy through enhanced metabolic fitness [59], greater anti-tumor activity [43,60], and greater radiation efficacy with low/no side effects in syngeneic mouse tumor models [61]. The SIRPα interaction surface of CD47 is evident in co-crystal structures (PDB: 2JJS, 2JJT) and, as described by Hatherley et al. [62], includes the region centered on the FG loop (Gly92 –Tyr 113) and N-terminal pyroglutamate of CD47 (**Fig 1**). By contrast, the interaction site of TSP1 on CD47 can only be inferred through biochemical studies defining the necessity of a heparan sulfate proteoglycan modification at CD47 Ser64 [63]. With this limited information, we infer that the binding sites of SIRPα and TSP1 do not overlap on CD47 [63]. It has been shown that biologics in clinical development bind CD47 and appear to sterically block binding of both ligands [64]. We hypothesize that chemical probes can be generated that block CD47 signaling in a ligand-selective manner by either targeting distinct sites on CD47 (while not sterically interfering with the second ligand binding site) or by individually targeting the CD47 ligands. Ligand-selective chemical probes are not currently available and would be valuable new agents to study CD47 function and mechanism of action *in vitro* and *in vivo*.

Our first goal is to discover small molecules that disrupt the CD47-SIRPα protein-protein interaction (PPI) and develop them into ligand-selective CD47 inhibitors for use as research tools and pretherapeutic lead compounds for numerous cancers. We leveraged the well-described biochemical characterizations of the soluble CD47-SIRPα interacting domains [62,65,66] as the basis for creating high throughput screening (HTS)-compatible PPI inhibitor assays. While libraries of PPI-focused chemical screening material continue to evolve [67–69], many HTS-compatible biochemical assays of PPIs have been reported with a range of commercially available reagents (as reviewed in [70,71]). Among these, time-resolved Förster resonance energy transfer (TR-FRET, HTRF[72]) and amplified luminescent proximity homogeneous assay (ALPHA) screens [73] are the most widely used for non-peptide-based PPI assays. They offer the ability to “mix and read” in a homogeneous format (no washing step required) that is compatible with a range of protein affinity tags and ultra-high density plates (1536 well). Such assays have been used successfully in the discovery of PPI inhibitors [71].

Herein, we describe the primary steps in this discovery and development project that involved the creation and validation of biochemical quantitative HTS (qHTS) assays capable of measuring CD47-SIRPα disrupting activity of large libraries of chemically diverse small molecules. We describe the properties of these assays that enable the efficient differentiation of active molecules from artifacts. We also provide a technology comparison for common commercial screening reagents employed in biochemical PPI assays to inform future screening campaigns. Lastly, the performance of the validated assays is evaluated in a large library, automated primary qHTS and follow-up study to show the configuration and utility of these assays in a discovery screening campaign.

## Methods

### Protein expression and purification

CD47-CD4-6His: CD47’s extracellular domain (Gln19—Pro139) was subcloned into the mammalian expression vector pTT3 to create a fusion with rat CD4 (domains 3 and 4) and a 6XHis affinity tag (herein referred to as CD47-CD4-6His). CD47 Cys33 was mutated to Gly to avoid protein aggregation [62]. Protein was expressed in expi293F cells using the expi293 expression system reagents (Thermo-Fisher). Protein was harvested and purified using immobilized metal-affinity chromatography (IMAC) followed by size-exclusion chromatography (SEC) with a final yield of 19 mg per Liter of cell culture. The purified protein was stored in 20 mM HEPES pH 7.3, 150 mM NaCl at a concentration of 52.5 μM.

SIRPα: the codon-optimized extracellular domain d1 of SHPS-1 isoform 1 (residues 31–149; UNIPROT P78324) with an N-terminal 6XHis-thioredoxin-SUMO-tag and a C-terminal biotin ligation sequence (Avi-Tag) was synthesized by BIOBASIC, Inc and cloned into the multi-cloning site 1 (MCS1) of the expression vector pCDF-Duet between the NcoI and EcoRI restriction sites. The protein was expressed in Origami B (DE3) cells (Novagen). Cells were cultured overnight at 37°C. The next day, protein expression was induced with 0.4 mM isopropyl 1-thio-D-galactopyranoside when *A*_*600*_ reached 1.0. Cells were cold shocked on ice for 1 hour and grown at 20°C for 24 h, then pelleted and frozen at -80°C until further use. To purify SIRPα-Avi, cell pellets were lysed in buffer A (20 mM Tris-HCl, pH 8.0, 0.2 M NaCl, 30 mM imidazole, 5% (v/v) glycerol), 15 units/ml benzonase (Sigma), 0.5 mg/ml lysozyme, protease inhibitor mixture (Roche Applied Science) via sonication. The clarified supernatant was then applied on a Nickel HisTrap affinity column (GE Healthcare), and the protein was eluted with a 3–100% gradient of buffer B (20 mM Tris pH 8.0, 0.4 M NaCl, 1 M imidazole, 5% (v/v) glycerol). The His-Trx-SUMO tag was removed by ULP1 cleavage (overnight, 4°C) in dialysis buffer (20 mM Tris pH 8.0, 30 mM NaCl, 1% (v/v) glycerol). The cleaved protein was subsequently biotinylated *in vitro* using BirA according to the manufacturer’s instructions (Avidity, Inc). The mixture was then passed on a Nickel HisTrap and QHP anion exchange chromatography columns to remove the cleaved tag, ULP1 and BirA. The final step was size exclusion on Superdex 75 in buffer C (20 mM HEPES pH 7.5, 0.15 M NaCl). Fractions containing SIRPα-biotin were identified by SDS-PAGE, pooled and concentrated. Aliquots at concentrations between 490 μM and 1.8 mM were flash-frozen in liquid nitrogen and stored at -70°C. The final yield was ~15–20 mg of pure SIRPα-biotin protein per Liter of cell culture. Non-biotin-labeled SIRPα (SIRPα-cold) was purified Nickel His-Trap and SEC yielding ~30–40 mg of pure protein per Liter of cell culture.

SIRPα-Fc: the extracellular domain (comprised of the d1, d2, and d3 domains) was cloned into the mammalian expression vector pFLAG-CMV1 to create a C-terminal fusion with human IgG Fc. The protein was expressed in HEK293T cells grown in DMEM with 1% BSA (no serum) and harvested between days 2 and 7 after transfection (Turbofect; ThermoFisher). The Antibody Purification Kit (PROSEP-G; Millipore) was used to purify SIRPα-Fc from the conditioned media to >95% purity. The final protein was dialyzed and stored at a concentration of 1.8 mg/mL in PBS buffer at -70°C.

### Surface plasmon resonance binding studies

The SIRPα and CD47 extracellular domain interaction was analyzed using the BIAcore 3000 system (BIAcore, GE) at 25°C. SIRPα-biotin was loaded onto channel 2 of a streptavidin (SAV)-functionalized CM chip (Xantec SCB SAHC 1000M) at 150 nM (PBS, 0.005% IGEPAL) with an injection time of 6 min and a flow rate of 10 μL/min to yield a response of 5416 RU. Response to CD47 binding was measured at a flow rate of 50 μL/min and an injection time of 2 min. The SIRPα surface was regenerated between CD47 concentration runs using 10 mM Glycine buffered to pH 2.0.

### Compound libraries

The 1,280 compound LOPAC (Library of Pharmacologically Active Compounds) was purchased from Sigma-Aldrich. The LOPAC library was supplied at 10 mM concentration in DMSO and arrayed into 1536-well microtiter plates (Greiner Bio-One 782855) in a serial 7-point dilution scheme (stock conc: 10 mM, 2.5 mM, 625 μM, 156 μM, 39 μM, 9.8 μM, 2.4 μM). Aluminum adhesive plate seals were applied with an Abgene Seal-IT 100 (Rochester, NY) plate sealer and plates were stored at ambient temperature in a desiccated dry-keep. The Genesis compound library (https://ncats.nih.gov/preclinical/core/compound/genesis) comprised 94,965 samples at the time of screening, and was screened in a serial 6 or 7-pt qHTS as above.

### qHTS assays

Reagents were diluted in room temperature assay buffer according to final concentrations in **Table 1,** and all manipulation/incubations were subsequently carried out at room temperature.

**Table 1:** Optimized assay conditions for 1536-well plate format.

Assay	CD47 conc	SIRPα conc	Acceptor & conc	Donor & conc	Buffer	Plate type
TR-FRET (CisBio)	12.5 nM	100 nM	SAV-XL665; 85 nM	Anti-6HIS-Tb cryptate; 0.7 nM	PBS, pH7.4; 0.005% IGEPAL CA-630; 0.1% BSA	1536 well; medium bind; white solid; high base; Greiner 789075
TR-FRET (LANCE)	12.5 nM	100 nM	SAV-APC; 26 nM	Anti-6HIS-Eu chelate; 3 nM		
AlphaScreen	12.5 nM	25 nM	NTA bead; 10 μg/mL	SAV bead; 10 μg/mL		

CD47-CD4-6His and the 6XHis affinity reagent were premixed and added sequentially with the remaining reagents using a BioRaptr Flying Reagent Dispenser (Beckman Coulter) in the volumes and sequence indicated in **Tables 2 and 3**. Compound was transferred to the assay plates using a 23 nL 1,536-pin array (Hornet PinTool; Wako Automation). Importantly, the compounds were added between the CD47 and SIRPα reagent additions to allow their equilibration with protein binding sites in advance of the PPI. The streptavidin reagent was always added as the final step as in manufacturer guidance and empirical observations of assay performance. Assay plates were then centrifuged at 1000 RPM for 30 sec then incubated for 30 min at room temperature protected by a gasketed stainless steel plate lid (Wako Automation). Final plate reads were performed on an EnVision Multimodal Plate Reader (PerkinElmer) according to the settings in **Tables 2 and 3**. DMSO-only control plates were tested as the first and last plates in the LOPAC library screen for a total of 9 assay plates and every 50 plates in the Genesis library screen. Controls were included in columns 1–4 of each plate along with DMSO (vehicle control). Column 1 contained the low signal control (-CD47; set as -100% activity), column 2 contained the neutral signal control (all assay components, no inhibitors; set as 0% activity), column 3 contained the inhibitor control at constant IC_50_-defined level (SIRPα-cold, 1 μM) and column 4 contained an inhibitor titration (SIRPα-cold, 10 μM– 30 nM). Plate data were normalized to column 1 and 2 signals (-100% and 0% activity, respectively).

**Table 2:** TR-FRET assay protocol.

Sequence	Parameter	Value	Description
1a	Reagent	3 μL	CD47-CD4-6His and Anti-6His Eu (LANCE) or Tb (CisBio) Donor reagent added to respective wells in Assay buffer; white/solid bottom high base, med bind 1536-well plates (Greiner 789075)
1b	Reagent	2 μL	Assay buffer negative control for background fluorescence
2	Reagent	23 nL	library compounds (7-pt, 1:4 intraplate titration series) or DMSO control transferred by Pin Tool
3	Centrifuge	30 sec	Centrifuge plate for 30 sec at 1000 RPM
4	Incubation	30 min	Incubate reagents at room temperature for 30 min, protected from light
5	Reagent	2 μL	SIRPα-biotin in Assay buffer
6	Incubation	30 min	Incubate reagents at room temperature for 30 min, protected from light
7	Reagent	1 μL	SAV-APC Acceptor reagent added to respective wells
8	Centrifuge	30 sec	Centrifuge plate for 30 sec at 1000 RPM
9	Incubation	30 min	Incubate reagents at room temperature for 30 min, protected from light
10	Detector	EnVision	Measure Top TR-FRET on EnVision (Eu/APC filter set); calculate ratio Ch1/Ch2
Notes			
1	Assay buffer composition		Phosphate buffered saline (PBS; 10 mM PO_4_^3−^, 137 mM NaCl, and 2.7 mM KCl; pH 7.4), 0.05% IGEPAL CA-630
10	Detector settings		Mirror = LANCE/DELFIA Dual Enh; Ex. Filter = UV2[TRF] 320; Em. Filter = APC 665; 2^nd^ Em. Filter = Europium 615; Measurement h = 6.5; Ex. light (%) = 100; Delay = 60 μs; Window t = 100 μs; No. Seq. windows = 1; Time between flashes = 2000μs; No. of flashes = 100; No. of flashes for 2^nd^ detector = 100; Calculation: “Ratio Ch1/Ch2”

**Table 3:** ALPHAScreen assay protocol.

Sequence	Parameter	Value	Description
1a	Reagent	2 μL	SIRPα-biotin added to respective wells in Assay buffer; white/solid bottom high base, med bind 1536-well plates (Greiner 789075)
1b	Reagent	2 μL	Assay buffer negative control for background fluorescence
2	Reagent	23 nL	library compounds (7-pt, 1:4 interplate titration series) or DMSO control transferred by Pin Tool
3	Centrifuge	30 sec	Centrifuge plate for 30 sec at 1000 RPM
4	Incubation	30 min	Incubate reagents at room temperature for 30 min, protected from light
5	Reagent	2 μL	CD47-CD4-6His added to respective wells in Assay buffer
6	Centrifuge	30 sec	Centrifuge plate for 30 sec at 1000 RPM
7	Incubation	30 min	Incubate reagents at room temperature for 30 min, protected from light
8	Reagent	1 μL	Ni-NTA Acceptor bead mix added to respective wells
9	Reagent	1 μL	SAV Donor bead mix added to respective wells
10	Centrifuge	30 sec	Centrifuge plate for 30 sec at 1000 RPM
11	Incubation	60 min	Incubate reagents at room temperature for 60 min, protected from light
12	Detector	EnVision	Measure Alphascreen on EnVision (1536A1 aperture)
Notes			
12	Detector settings		Aperture = 1536 plate HTS AlphaScreen; plate-detector distance = 0; Total measurement time = 240 μs; Ex. time = 80 ms (33%); Afterglow CT1 corr. factor = 0; Glow CT2 corr. factor = 0; Bleach CT3 corr. factor = 0; Ref. signal= 417733; Ref. AD gain = 2

### Data analysis

Signal to background (S/B) was calculated as mean specific signal/mean background signal. Percent corrected values (%CV) for the positive and negative controls were calculated as mean signal/standard deviation. Z’ was calculated from positive and negative controls as in Zhang et al. [74]. Concentration-response curves (CRC) from the qHTS were determined from the normalized and corrected data as previously described [75]. CRCs for each compound were curve-fitted and categorized into four major curve classifications (cc) as described [76], using open access NCATS software (http://ncgc.nih.gov/pub/openhts/curvefit/). The CDD Vault software platform was subsequently used for data sharing and cross assay comparisons.

### Database deposition

The LOPAC data generated in this study has been deposited in PubChem (https://pubchem.ncbi.nlm.nih.gov/classification/#hid=1), use “keyword” = AID in the pulldown menu. The CD47-SIRPα protein-protein interaction—AlphaScreen assay qHTS validation PubChem AID is 1347059. The CD47-SIRPα protein-protein interaction—LANCE TR-FRET assay qHTS validation PubChem AID is1347057. The CD47-SIRPα protein-protein interaction—CisBio TR-FRET assay qHTS validation PubChem AID is 1347058.

## Results

### Design and preparation of SIRPα and CD47 soluble domains

To recapitulate the biological interaction of CD47 and SIRPα, we designed expression constructs to produce their recombinant, soluble, extracellular interaction domains. CD47-CD4-6His was produced in a mammalian expression system and was previously shown to bind recombinant SIRPα [77]. As expected, recombinant CD47-CD4-6His was highly glycosylated, migrating as a diffuse band at ~60 kDa, as compared to the molecular weight of 40 kDa predicted from the protein sequence (**S1 Fig**). The heterogeneity in glycosylation of CD47 has no reported effect on its ability to bind SIRPα [78].

Non-glycosylated SIRPα binds CD47 with high nM affinity [78]. However, recombinant SIRPα containing abnormally high and heterogeneous glycosylation showed impaired CD47 binding [79]. To reduce biological assay variability, we chose to produce SIRPα in a glycosylation-deficient system. SIRPα-Avi (Isoform 1 allele 1) is expressed in *E*. *coli* as a fusion construct that comprises an N-terminal purification tag followed by the extracellular CD47-binding fragment of SIRPα linked to a C-terminal Avi-Tag. After affinity capture and tag cleavage, SIRPα-Avi was biotinylated *in vitro* (SIRPα-biotin) using purified recombinant *E*. *coli* biotin ligase (BirA) to >98% incorporation, as measured by mass spectrometry (**S2 Fig**). The purified protein is monomeric as judged by SEC. Small-angle x-ray scattering (SAXS) studies (see **S1 File**) confirmed that SIRPα is monomeric in solution (up to at least 1.8 mM), with the C-terminal Avi-tag positioned away from the CD47-binding region, demonstrating its suitability for our assay (**S3 Fig)**.

**Fig 2:**
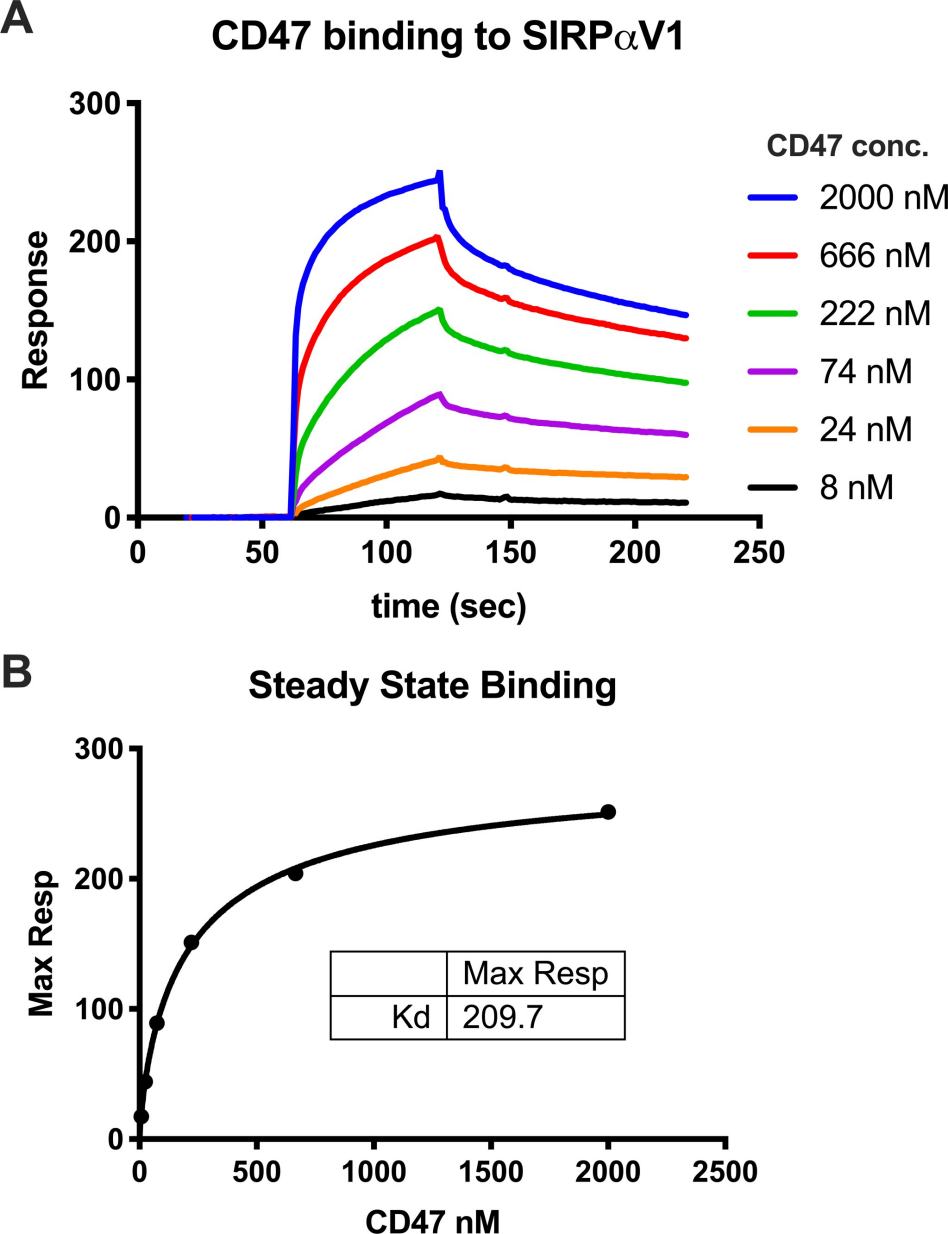
Validation of recombinant construct binding affinity by SPR. SIRPα-biotin was immobilized on a SAV-chip followed by injection of CD47-CD4-6His at varying concentrations. (A) The association and dissociation responses are shown as an overlay for each concentration tested. (B) The maximum response of the association phase was plotted against concentration and fitted to a steady-state binding curve to approximate the K_d_ of the interaction pair.

To validate the biologically relevant interaction between our PPI reagents, we used SPR spectroscopy to measure their steady state binding affinity (**Fig 2**). SIRPα-biotin was immobilized on a SAV-chip with CD47-CD4-6His injected at multiple concentrations to yield a K_d_ of approximately 200 nM. This is comparable to a previous report of 300 nM and 279 nM employing similar constructs and measurement via SPR [66,80] and 470 nM for soluble CD47 binding to SIRPα-expressing CHO cells measured by fluorescence intensity [81].

### Assay development

**Fig 3:**
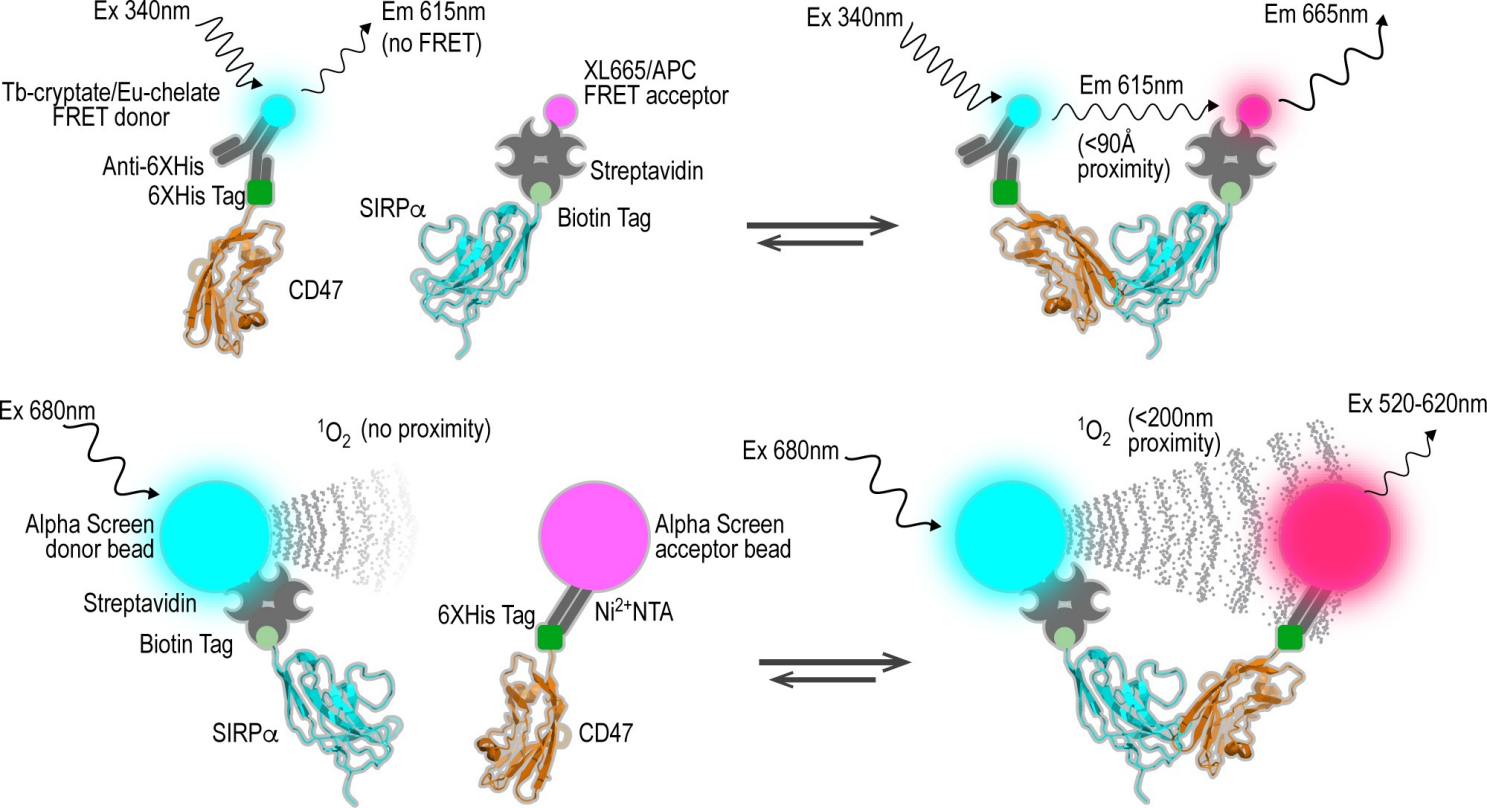
Schematic for qHTS assays. TR-FRET based assays (upper panel) and ALPHAScreen assay (lower panel). CD47 and SIRPα represented as their x-ray crystal structures from PDB 2JJS, chain D and chain B, respectively.

**Fig 4:**
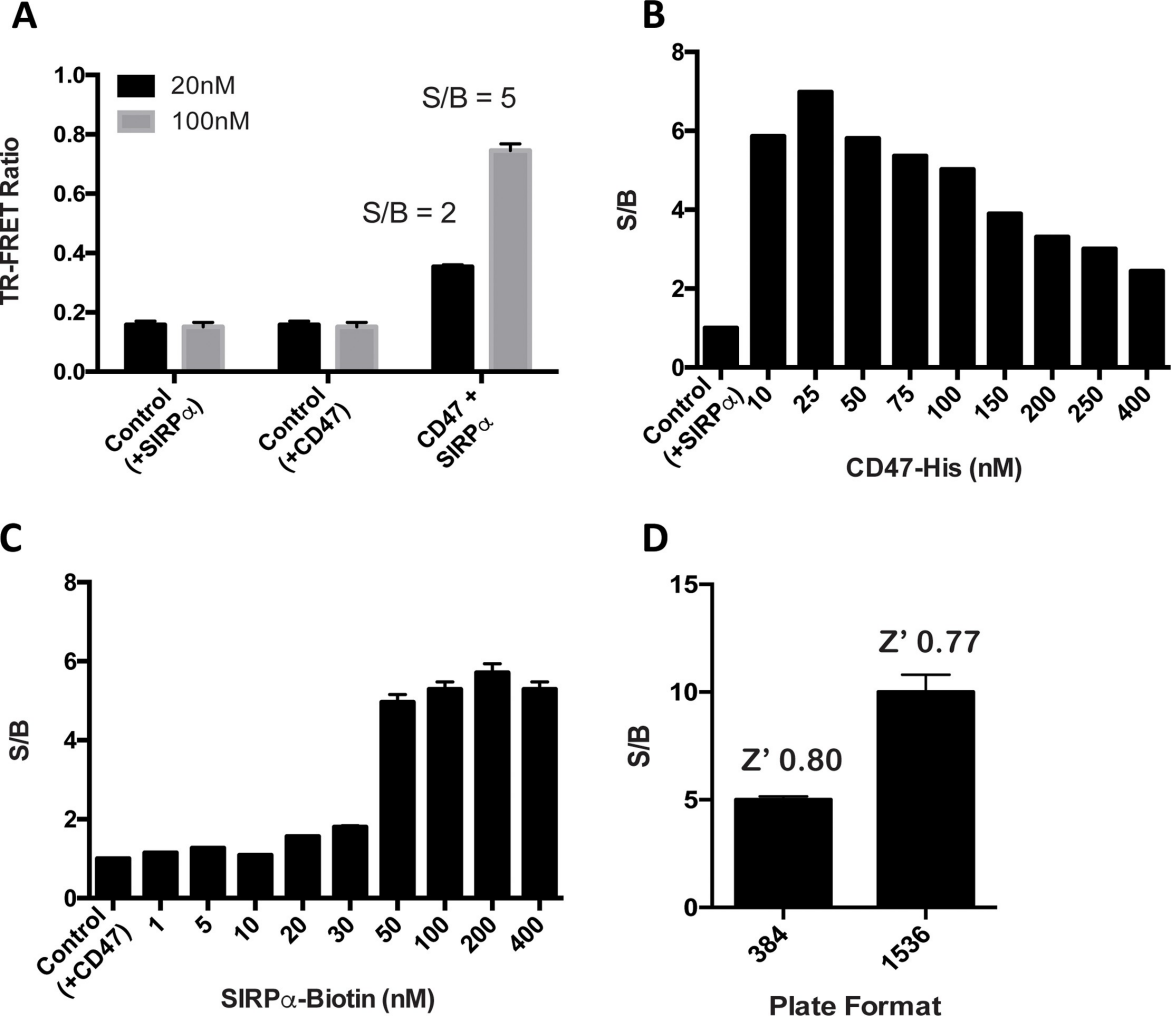
Optimization of CisBio TR-FRET assay. (A) Initial assay feasibility performed in 384-well plate format using CisBio TR-FRET reagents. CD47 and SIRPα were tested at 2 concentrations (20 nM and 100 nM), and the FRET ratio of the complete reaction was compared to controls lacking either CD47 or SIRPα to calculate the S/B (n = 16). (B) CD47 or (C) SIRPα concentrations were titrated while the other was fixed as indicated in (A) in 384-well plates to find their optimal assay concentrations as judged by S/B (n = 4). (D) Assay performance in 384-well or 1536-well formats compared using optimized concentrations derived from panels B and C. Assay Z’ indicated for each plate type (n = 32).

We developed a TR-FRET assay to quantitatively measure the interaction of CD47 and SIRPα [82]. We started with the TR-FRET reagents from CisBio for feasibility and optimization studies. The donor reagent consists of an anti-6His monoclonal antibody tagged with a Terbium cryptate (61HI2TLA) to provide a long-lived fluorescent signal bound to the 6His tag of CD47 (**Fig 3A**). The acceptor consists of streptavidin tagged with an XL665 fluorochrome (SAV-XL665; 610SAXLA) to associate with the biotin tag on SIRPα. Feasibility testing was conducted in 384-well format to assess signal to background and assay specificity. Using the experimentally determined K_d_ as a starting point (see **Fig 2**), CD47 and SIRPα concentrations of 100 nM resulted in a S/B of 5 compared to the same mixture lacking CD47 (**Fig 4A**). To find the optimal concentration of protein reagents for each assay, each component was titrated to saturation (**Fig 4B and 4C**). We then miniaturized the assay volumes for use in 1536-well format and proceeded to test feasibility and further optimize the assay (**Fig 4D**). Finally, using the adjusted ligand protein concentrations, we optimized assay buffer, donor and acceptor levels, plate type, order of reagent addition, and incubation time (**Table 1 and S4 Fig**). Briefly, we found that nM concentrations of CD47 (12.5 nM) and SIRPα (100nM) in PBS buffer with minimal detergent (0.005% IGEPAL CA-630) and BSA (0.1%) produced a robust assay.

Second, we developed the LANCE (LANthanide Chelate Excite) TR-FRET assay, which consists of an anti-6His monoclonal antibody linked to an Eu-chelate (Anti-6His-Eu; AD0110) for the donor associated with the CD47 reagent and streptavidin linked to allophycocyanin (SAV-APC; AD0201) as acceptor associated with SIRPα. Using the optimized CD47 and SIRPα concentrations from the CisBio assay optimization as a starting point, the LANCE assay reagents were titrated in 1536-well format to arrive at a S/B of 12 (**Table 4 and S5 Fig**) using optimal conditions outlined in **Table 1**. As with the CisBio assay (**S4 Fig**), the endpoint is stable (≥80% of initial signal) for at least 48 h (**S6 Fig**).

**Table 4:** Optimized assay performance in 1536 well plate format.

Assay	S/B	%CV (neg.)	%CV (pos.)	Z’	Read time
TR-FRET (CisBio)	8.4	6%	13%	0.79	14 min
TR-FRET (LANCE)	12.1	5%	8%	0.85	14 min
AlphaScreen	75	14%	7%	0.79	9 min

Third, we developed the AlphaScreen assay as a cross validation orthogonal assay. It uses a bead-based luminescent oxygen channeling [83] technology based on the transfer of energy from the laser-excited (680 nm) donor bead to acceptor beads in the form of singlet oxygen to produce a luminescent signal (520–620 nm) [84,85]. Singlet oxygen has a limited lifetime (4 μsec half-life) prior to relaxing back to ground state during which it can diffuse approximately 200 nm in solution. If an acceptor bead is within that distance, energy is transferred from the singlet oxygen to thioxene derivatives within the acceptor bead, subsequently culminating in light production at 520–620 nm. In the absence of an acceptor bead, singlet oxygen relaxes to ground state and no signal is produced. This proximity-dependent chemical energy transfer is the basis for ALPHAScreen's homogeneous format (**Fig 3**).

The ALPHAScreen assay was optimized using the CD47 and SIRPα concentrations from the CisBio assay development as a starting point. While the optimal CD47 concentration remained unchanged throughout the three assays, the optimal SIRPα concentration is 4-fold lower for the ALPHAScreen assay, possibly due to the differences in SAV-bead-binding capacity versus streptavidin in solution. Optimization also allowed for a 2-fold lower bead concentration than suggested by the manufacturer, reducing the cost per data point [86]. The resulting ALPHAScreen assay yielded robust HTS parameters (Z’ of 0.79, S/B = 75; **Table 4**), a slightly faster read time than the TR-FRET assays (9 min/plate vs. 14 min/plate), and a common assay buffer and plate type with the CisBio and LANCE assays (**Table 1**).

### Assay validation

**Fig 5:**
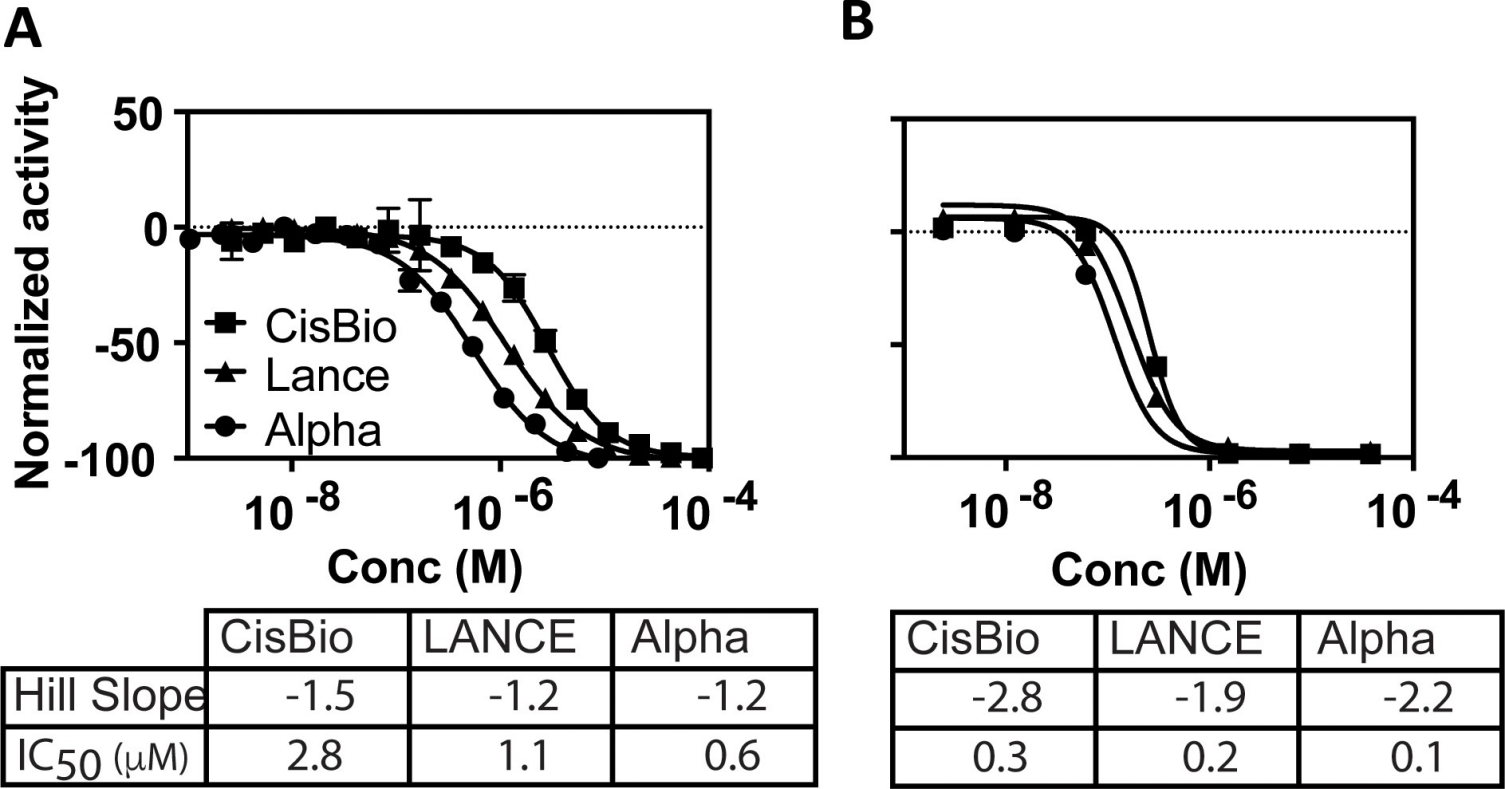
Validation of assay specificity and performance. (A) Assay specificity was validated using SIRPα-cold titrated to inhibit the CD47-binding activity of SIRPα-biotin in the screening assays. Hill slope and IC_50_ are indicated (n = 4). (B) Assay performance using a positive control small molecule (biotin) was assessed in all three assays (n = 1). Normalized activity calculated with neutral control (+CD47, +SIRPα, no inhibitor) as 0% and low control (-CD47 +SIRPα, no inhibitor) as -100% activity. Assay activity with tested agents was then compared and normalized to these controls.

We evaluated the performance of all three assays in a qHTS format using SIRPα-cold (non-biotinylated) as a specific inhibitor of SIRPα-CD47 interaction. The IC_50_ values exhibited good agreement with those obtained from the TR-FRET assay (2.8 μM and 1.1 μM for CisBio and LANCE, respectively) and the ALPHAScreen assay (0.55 μM; **Fig 5A**). Hill slopes indicate a near ideal bimolecular interaction (1.2–1.5, **Fig 5A**). We also evaluated the ability of a small molecule to act as an inhibitor in the assay, as the eventual goal of the HTS assay will be to screen large chemical libraries for small molecule antagonists of the protein-protein interaction between SIRPα-CD47. As there are no known small molecule inhibitors of this interaction, we chose to use free biotin to inhibit the interaction between SIRPα and the streptavidin-conjugated assay reagents. Free-biotin was added in advance to the SIRPα-CD47 complex prior to the addition of the streptavidin-conjugated reagent to overcome the extraordinarily slow off-rate of the complex once formed [87]. The activity of biotin was similar for all assays and exhibited the same inter-assay potency ranking as the large molecule SIRPα-cold (AlphaScreen>LANCE>CisBio; **Fig 5B**). The IC_50_ values of 260, 170, 115 nM were comparable to results generated by Perkin Elmer for similar assays evaluating the interference of biotin in assay media [88]. The cooperativity indicated by the Hill slope of between 2 and 3 is likely due to the multivalency of streptavidin and reflects the need to block multiple sites before the proximity signal is eliminated. This contrasts with SIRPα-cold’s interaction with the bivalent CD47-CD4-6His/anti-His antibody shown above. Assay robustness indicated by Z’ factor was “excellent” [74] (see **Table 4**) and allowed for advancement to pilot screening using the 1280 compound LOPAC Library (Sigma).

### Pilot screening assay comparison

**Fig 6:**
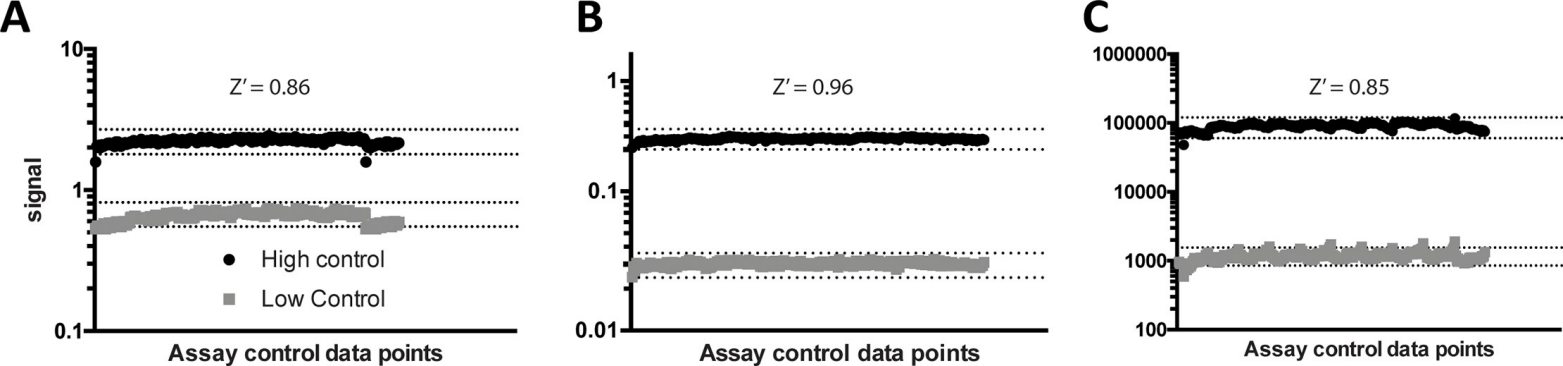
High and low control signal performance in pilot screening. Results for the (A) CisBio, (B) LANCE, and (C) AlphaScreen assays shown. Individual signal data points from neutral control (+CD47, +SIRPα, no inhibitor) and low control (+CD47 -SIRPα, no inhibitor) plotted by well for each plate in the 10-plate pilot screening run (n = 32 points per plate). Dotted lines represent + and– 20% of average signal for each high and low control. The Z’ factor was calculated from the high and low controls on each plate and averaged across all plates.

**Fig 7:**
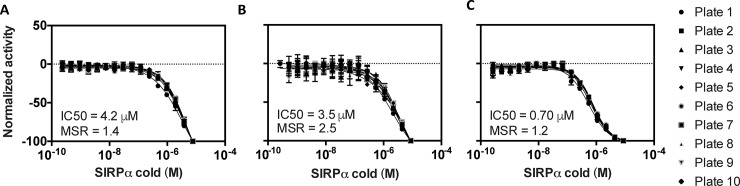
Control inhibitor performance in pilot screening. Results for the (A) CisBio, (B) LANCE, and (C) AlphaScreen assays shown. SIRPα-cold was titrated as a control inhibitor in each assay (n = 2). Error bars represent the standard deviation of two replicates. The average IC_50_ and minimum significant ratio (MSR) was calculated over the 10-plate run for each screening assay. MSR is defined as the smallest ratio between the potencies of two compounds that is statistically significant and is calculated as MSR = 10^2√2s^, where s is an estimate of the standard deviation of a log potency for one compound.

The LOPAC library was tested in qHTS format using a 7-point inter-plate titration spanning 38 μM to 2.4 nM (final concentration) in 1536-well format. All assays displayed robust performance as judged by Z’ factor, a statistical measure of the separations of the positive and negative control distributions. The Z’ was determined using column 1 and 2 of each plate as positive and negative controls, respectively (**Figs 6 and 7**).

**Fig 8:**
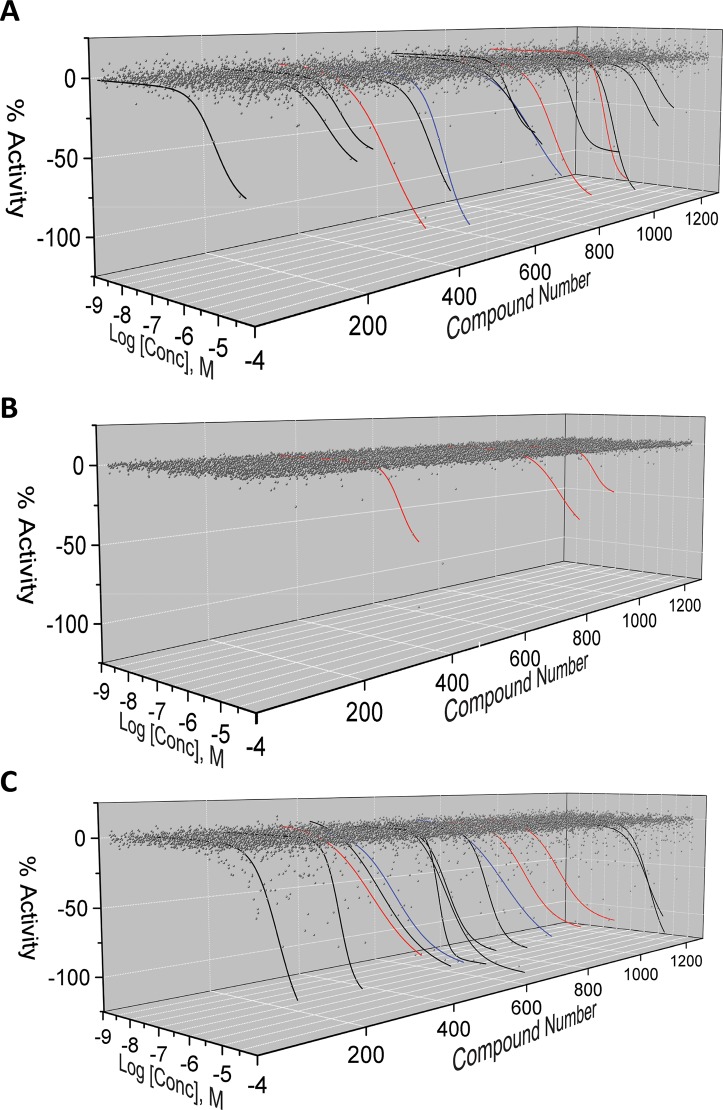
qHTS activity plots for LOPAC compounds. Results shown using (A) CisBio, (B) LANCE, and (C) AlphaScreen assays. Concentration response curves fit to data of active compounds using a 4-parameter logistic equation are included with solid lines. Black lines indicate activity only within the indicated assay. Blue lines indicate activity in both CisBio and AlphaScreen assays. Red lines indicate activity in all three assays.

Activity of library compounds was analyzed according to the curve classification (cc) as previously described [89,90]. Briefly, compounds were considered active when they displayed either concentration-dependent inhibition with two asymptotes and incomplete activity (-50–90%; cc -1.2), complete activity at the highest tested concentrations (<-90%; cc -2.1), or complete inhibition with two asymptotes indicating a saturable effect (<-90%; cc -1.1), as shown in **Table 5**. The highest overall activity was demonstrated in the AlphaScreen (17 out of 1280; 1.3%; **Fig 8**) followed by the CisBio TR-FRET assay (15 out of 1280; 1.2%) and the LANCE TR-FRET assay (3 out of 1280; 0.2%).

**Table 5:** Classification of active compounds from LOPAC screening.

Assay	cc -1.1	cc -1.2	cc -2.1	Total	After Counter Screen
TR-FRET (CisBio)	1	0	14	15	0*
TR-FRET (LANCE)	0	0	3	3	0*
AlphaScreen	8	3	6	17	5^#^

“cc” indicates curve classification for the fitted concentration response curves for each tested compound.

*TR-FRET donor channel activity

^#^AlphaScreen TruHits activity

We further characterized activity of these compounds with additional assay readouts, counter-screening assays, data characteristics, and reported compound promiscuity. Assay interference by chemical matter can take many forms in biochemical HTS assays [91,92] and can be mediated by compound fluorescence or aggregation, signal attenuation by absorbance, or singlet oxygen quenching and affinity tag-disruption in the case of the AlphaScreen.

**Fig 9:**
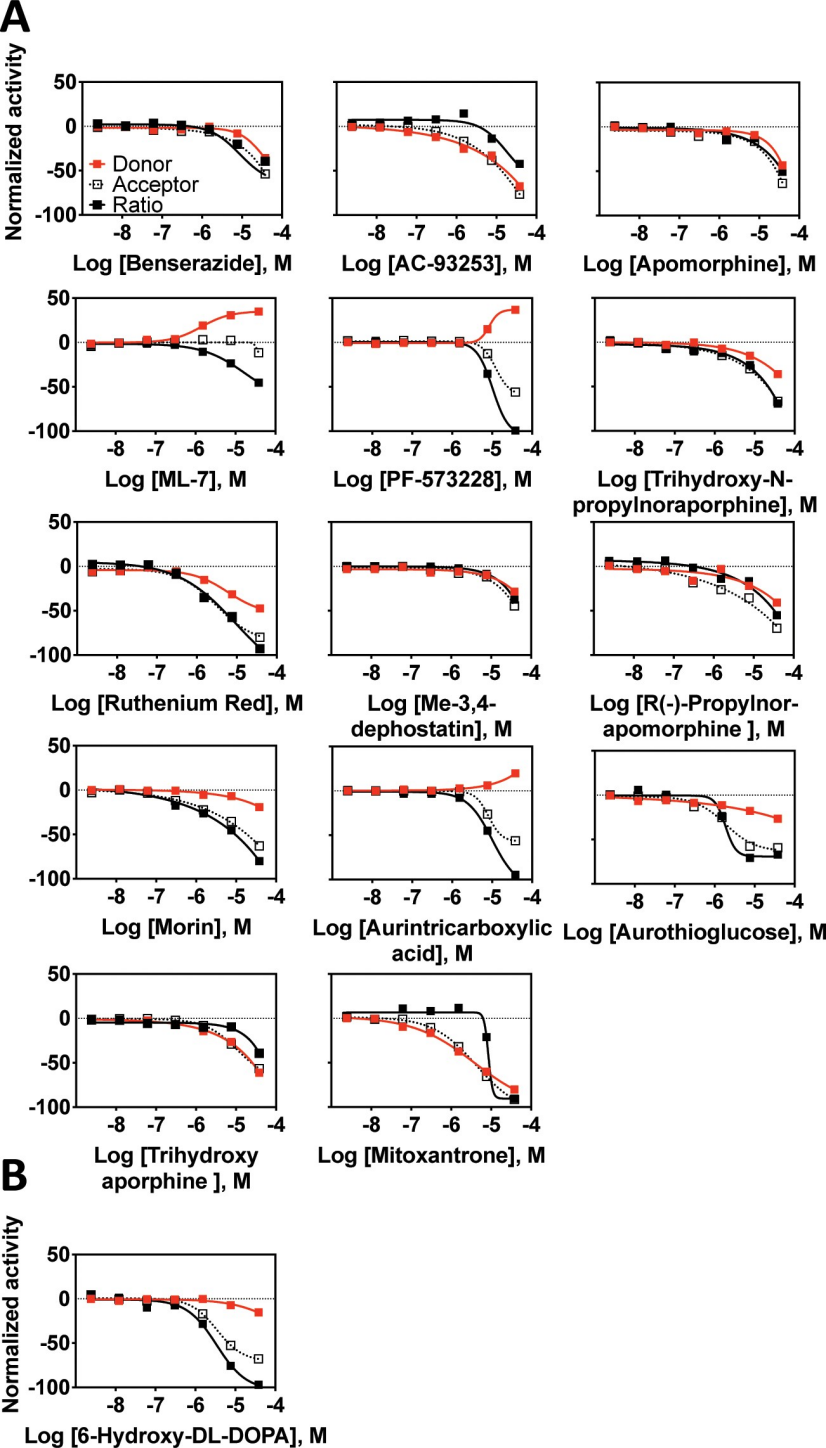
LOPAC active compounds from the CisBio TR-FRET assay. Normalized activity signal for the donor and acceptor channels and calculated ratio signal for each active compound reported for the 7-point concentration response generated using qHTS. Normalized activity was calculated with neutral control (+CD47 +SIRPα, no inhibitor) as 0% and low control (+CD47 -SIRPα, no inhibitor) as -100% activity. Assay activity with tested compounds was then compared and normalized within this range. (A) Compounds that interfered with donor fluorescence indicated by activity in the donor channel. (B) Reactive compound active in a variety of other unrelated screening assays.

**Fig 10:**
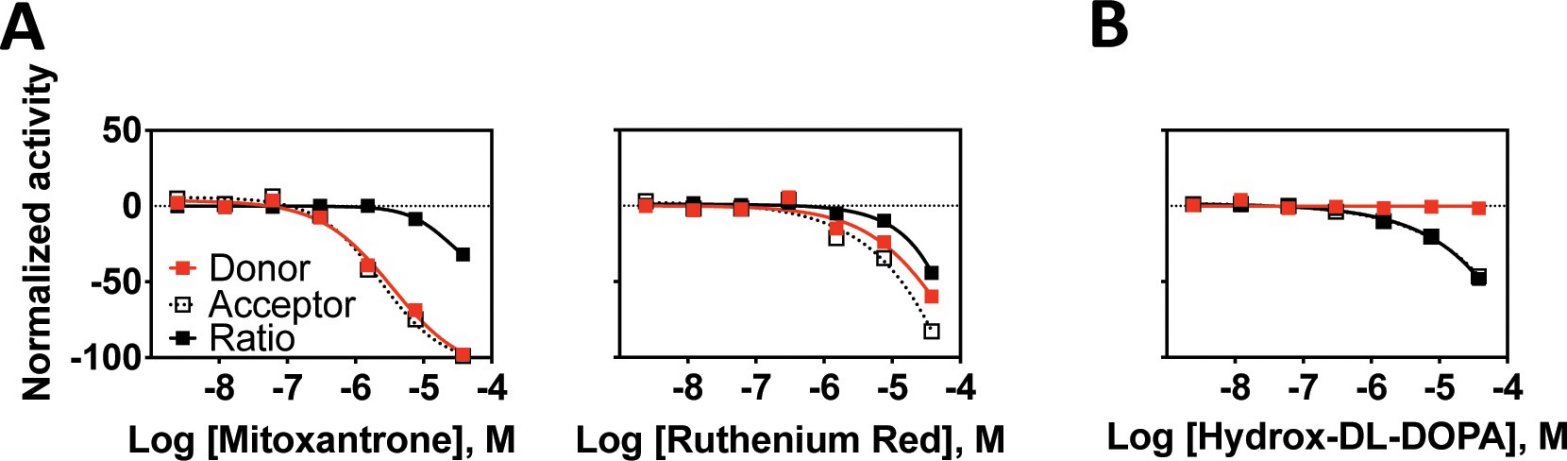
LOPAC active compounds from the LANCE TR-FRET assay. Normalized activity signal for the donor and acceptor channels and calculated ratio signal for each active compound reported for the 7-point concentration response generated using qHTS. Normalized activity was calculated with neutral control (+CD47 +SIRPα, no inhibitor) as 0% and low control (+CD47 -SIRPα, no inhibitor) as -100% activity. Assay activity with tested compounds was then compared and normalized within this range. (A) Compounds that interfered with donor fluorescence indicated by activity in the donor channel. (B) Reactive compound active in a variety of other unrelated screening assays (Pubchem).

**Fig 11:**
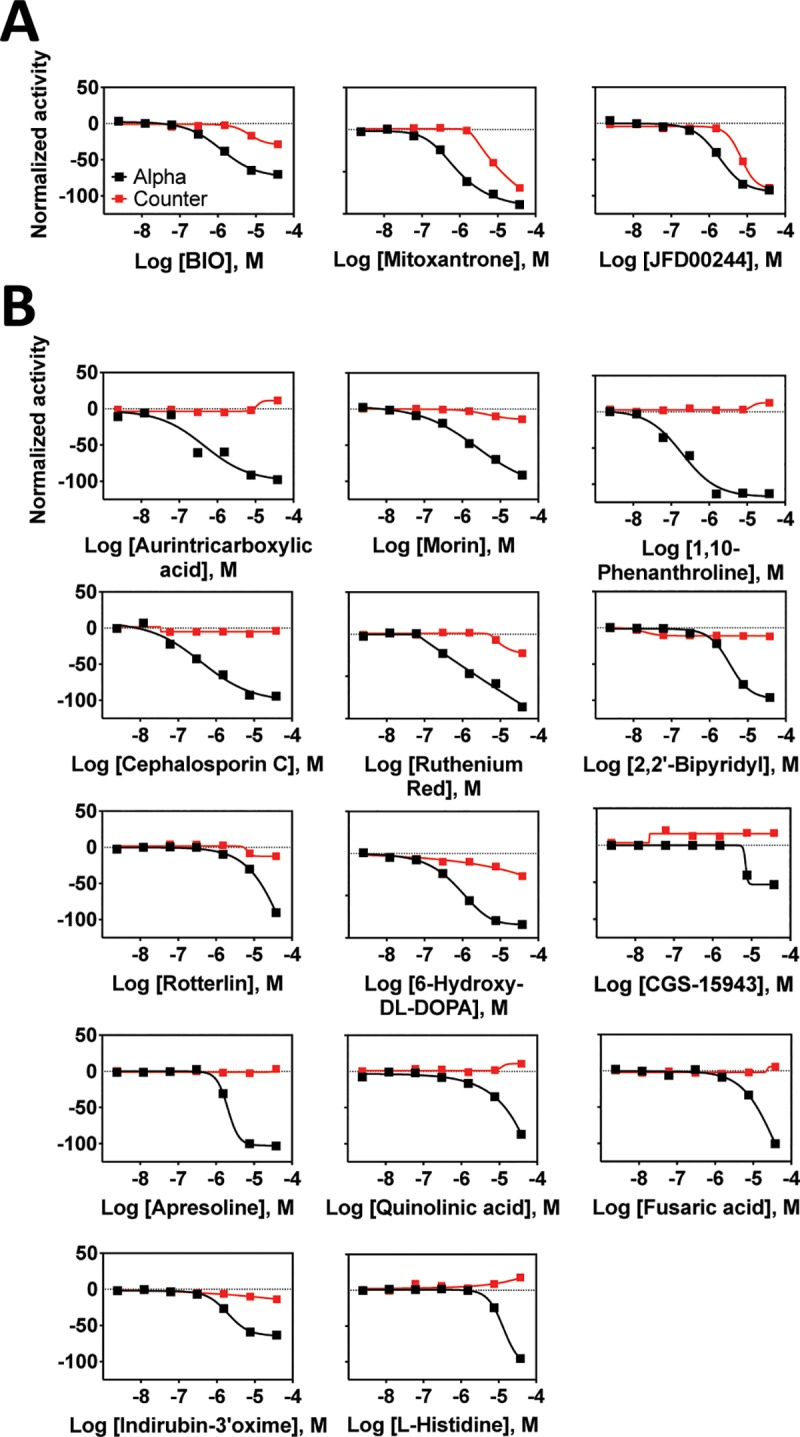
LOPAC active compounds from the AlphaScreen assay. Normalized activity signal for the specific AlphaScreen assay (Alpha) and the non-specific counter screen assay (counter) for each active compound reported for the 7-point concentration response generated using qHTS. Normalized activity in the AlphaScreen assay was calculated with neutral control (CD47 + SIRPα, no inhibitor) as 0% and negative control (CD47—SIRPα, no inhibitor) as -100% activity. Normalized activity in the counter screen assay was calculated with neutral control (+TruHits reagent, no inhibitor) as 0% and negative control (-TruHits reagent, no inhibitor) as -100% activity. Assay activity with tested compounds was then compared and normalized within this range. (A) Compounds that interfered with the assay as indicated by significant activity in the counter screen assay. (B) Reactive compounds identified as active in a variety of other unrelated screening assays.

In the TR-FRET assays, fluorescent compounds or attenuators were identified using an additional readout of donor channel fluorescence data. Changes in donor fluorescence that mirror or contribute to overall activity (ratio of FRET/donor) indicate assay interference and these compounds were removed from further consideration [93]. Donor channel artifacts were observed for 14 of the 15 CisBio assay active molecules (**Fig 9**), and for 2 of the 3 LANCE actives (**Fig 10**), resulting in 6-hydroxy-DL-DOPA as the only remaining candidate in both TR-FRET assays, which is known to have activity in a number of unrelated assays and is considered a promiscuous molecule. For AlphaScreen-active compounds, we employed an additional counter-screen assay (TruHits, Perkin Elmer) where the tagged SIRPα and CD47 interaction pair was replaced by a single dual-tagged peptide (Biotin-peptide-6XHis). Compounds displaying activity in this counter-screen do not specifically disrupt the SIRPα-CD47 interaction but are rather affinity tag disruptors, singlet oxygen or fluorescence quenchers, or possibly protein aggregators. Accordingly, 3 of 17 active molecules displayed counter-screen activity and were removed from further consideration (**Fig 11**).

The remaining active molecules were further scrutinized for non-ideal behavior that would indicate a mechanism other than CD47-SIRPα antagonism. A now well-recognized mechanism of assay interference involves molecules that tend to aggregate in aqueous assay buffers and adsorb assay reagents to result in apparent inhibitory activity [94–96]. As this phenomenon occurs at a threshold where the test molecule reaches its solubility limit, the concentration response curves will exhibit a steep Hill slope near the solubility limit [94]. Hill slopes are then an indicator of non-specific activity, and compounds were flagged as non-ideal inhibitors when they displayed Hill slopes outside of the -0.5 to -1.5 range, with the understanding that this does not definitively identify problematic molecules as aggregators [97]. To avoid compound aggregation and associated assay interference, all primary assays and follow-up tests were run with detergent present [97,98].

In the case of the lone active molecule in the TR-FRET assays (6-hydroxy-DL-DOPA), its Hill slope falls within tolerance for both the CisBio and LANCE assays. Of the AlphaScreen actives, aurintricarboxylic acid, cephalosporin C, Apresoline, Indirubin-3'-oxime, 2,2'-Bipyridyl, CGS-15943, L-Histidine hydrochloride, and ruthenium red all displayed Hill slopes outside of our acceptance criteria and were discarded. The remaining actives (morin, rotterlin, 1,10-Phenanthroline monohydrate, Quinolinic acid, Fusaric acid, and 6-hydroxy-DL-DOPA) have PAINS characteristics ([91,99,100] and the ChEMBL database) that would make them likely false positives and unsuitable for follow-up. 6-hydroxy-DL-DOPA is also suspected of assay interference due to its aggregator activity (see above) and in other studies [101], PAINS characteristics (as defined in the ChEMBL database), known redox reactivity, and assay promiscuity (active in 119 of 386 assays in Pubchem [102] at the time of manuscript preparation). Despite its well-recognized assay interfering activity, the activity of 6-Hydroxy-DL-DOPA in our assays was consistent with a well-behaved hit compound, and it was also the only consistently active molecule in all qHTS assays. It was thus selected for follow-up testing.

**Fig 12:**
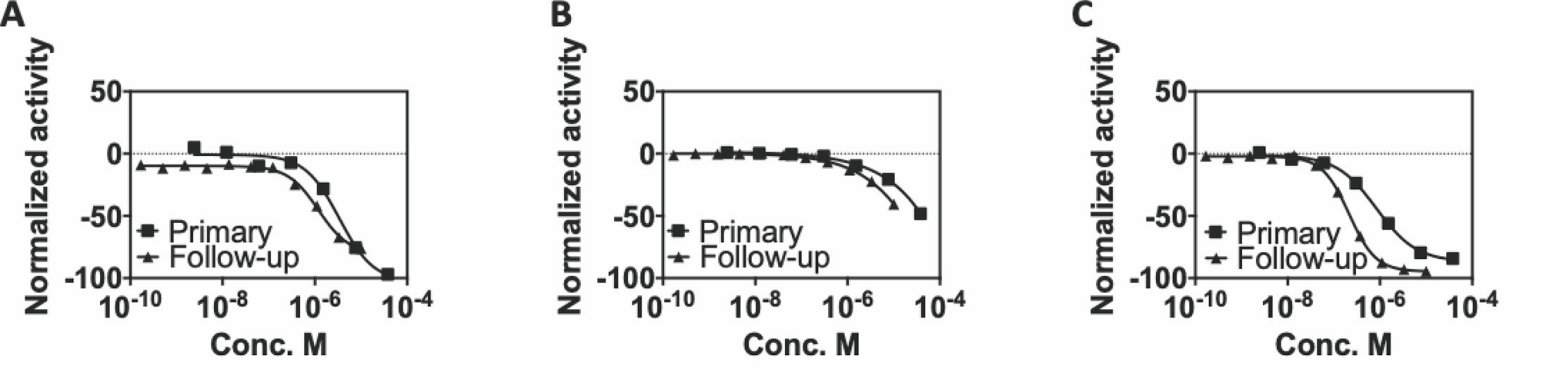
6-Hydroxy-DL-Dopamine follow-up testing comparison to primary screening. Results shown for the (A) CisBio, (B) LANCE, and (C) AlphaScreen assays. Follow-up testing was conducted using freshly plated compound from powder stocks in an 11-point concentration response (n = 1).

A fresh powder sample of 6-hydroxy-DL-DOPA was acquired from the supplier and authenticated via HPLC-MS from a 10 mM DMSO stock. Follow-up testing in the qHTS assays resulted in similar if not slightly more potent activity (**Fig 12**; CisBio and AlphaScreen). The compound was then evaluated for SIRPα-CD47 disrupting activity in the previously described SPR assay (vide infra) but failed to inhibit the protein-protein interaction under conditions similar to those of the qHTS assays.

**Fig 13:**
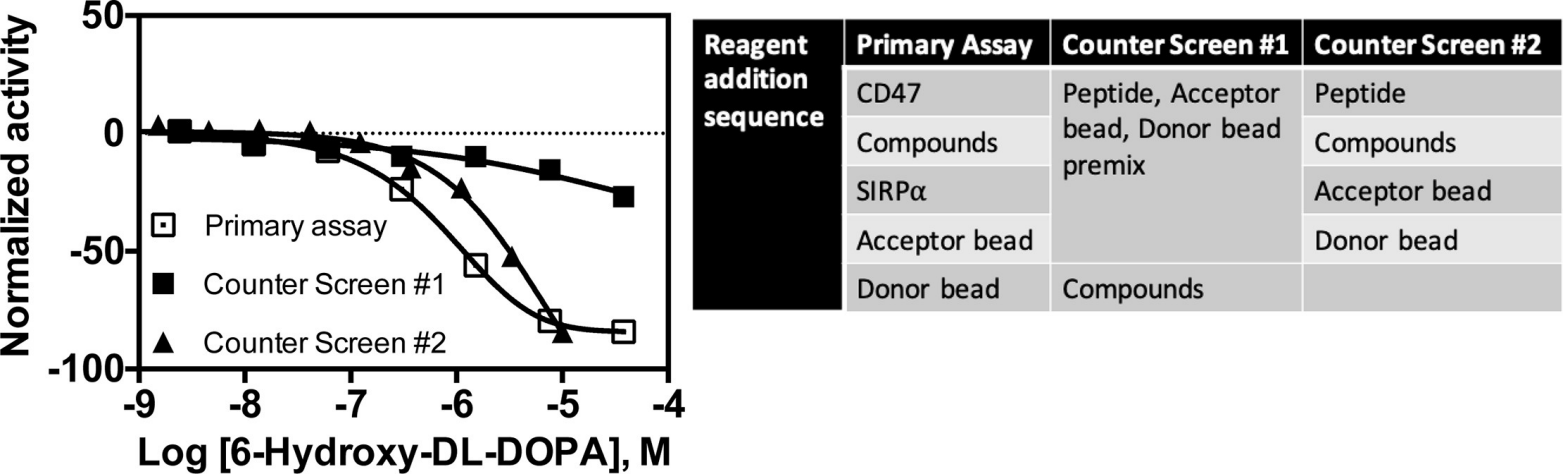
Comparison of 6-Hydroxy-DL-DOPA AlphaScreen. Activity shown using the CD47-SIRPα PPI assay (left panel) and the counter screen assay run under different reagent addition sequences (right panel).

As mentioned above, 6-Hydroxy-DL-DOPA is a known aggregator. This type of interference should lead to activity in the AlphaScreen counter screen assay. However, we found none in our assay, following the manufacturer’s protocol. In this experiment, the control peptide is premixed with both the acceptor and donor beads to form the complex and subsequently added to assay wells followed by the compounds. Under these conditions, the preformed bead-peptide complex may be kinetically and physically insensitive to aggregated compound. By comparison, in the primary assay, reagent proteins are exposed to the aggregated compound before the addition of beads. To unambiguously determine whether 6-Hydroxy-DL-DOPA showed interfering activity in the counter screen, we changed the protocol to more closely match the primary assay. Under these revised conditions (counter screen v.2), the peptide is added first to the wells, followed by the compound, then the beads (**Fig 13**). The revised conditions revealed activity in the counter screen assay v.2 that was similar to the primary screen assay and identified 6-Hydroxy-DL-DOPA as an interfering molecule. Therefore, it was eliminated from further consideration. In conclusion, no compounds were identified as specific inhibitors for the CD47-SIRPα interaction from the pilot screening study. However, we developed assays and counter assays that excluded many interfering compounds and that can be applied to larger chemically diverse collections and screening campaigns.

### Assay performance in fully-automated large library qHTS

**Fig 14:**
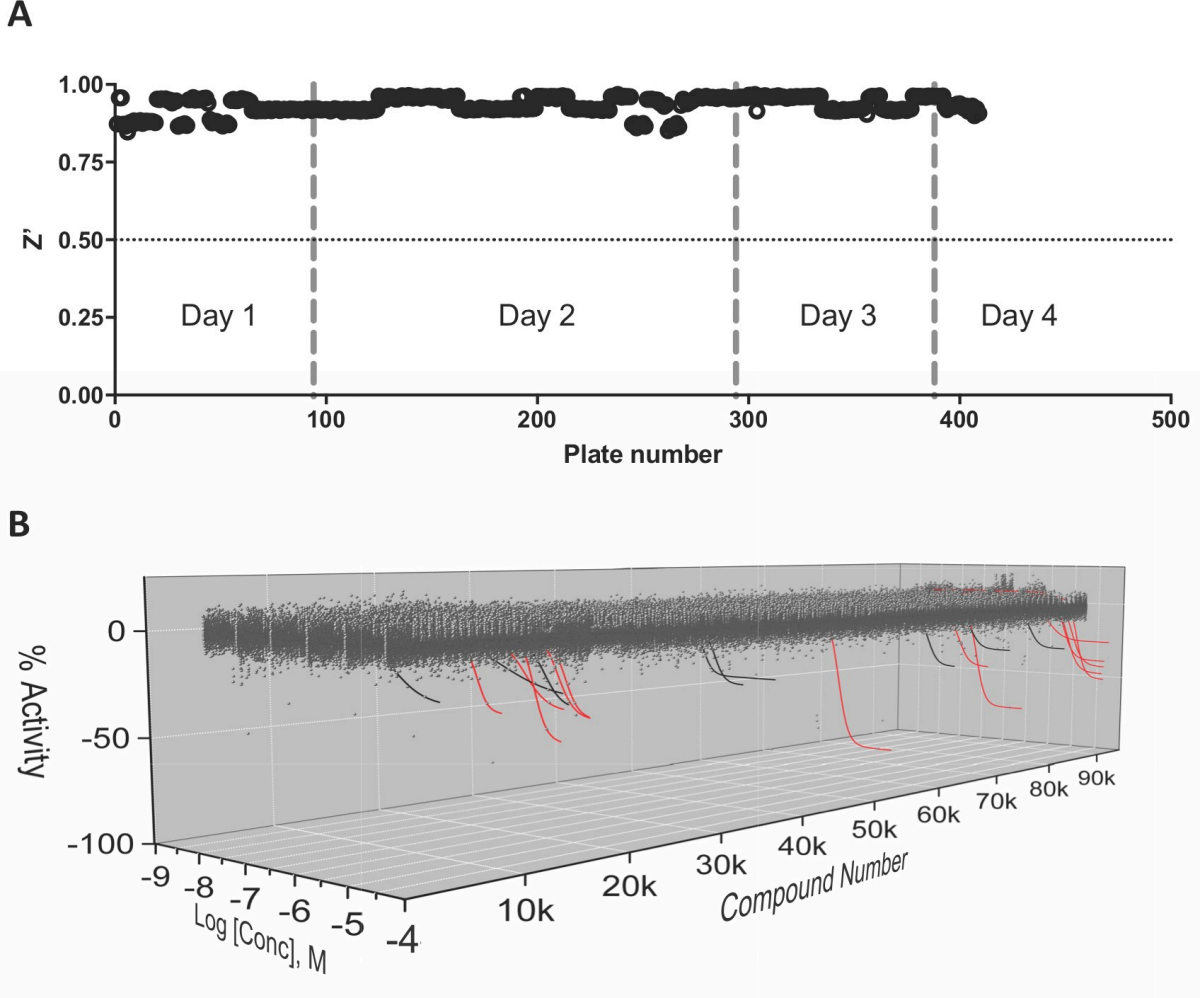
qHTS screen of the NCATS Genesis library (94,965 compounds) using the LANCE TR-FRET assay. (A) Z’ factor calculated from the intraplate controls. (B) Normalized activity of all compounds tested in a 6-point concentration response. Rendered curves represent all compounds displaying an activity with negative curve class. The curves rendered in red have a maximum activity ≤-25%.

We tested the performance of the above assays positioned as part of a large library, fully-automated, qHTS campaign. We used a primary screen with downstream counter and orthogonal screening assays capable of identifying assay artifacts from compounds active in primary screening. For the primary screen, we chose the LANCE TR-FRET assay as it had the best Z’ factor and lowest compound interference profile during pilot screening (**Fig 14**). As a counter screen we utilized the LANCE TR-FRET assay with a 6XHis-biotin peptide in place of the SIRPα-CD47 interaction (as in the AlphaScreen counter screen assay described above). This allowed for assessment of assay-interfering compounds with the same TR-FRET reagents as the primary screen. Orthogonal screening was used to confirm active compounds and was performed using the AlphaScreen SIRPα-CD47 interaction assay as described above. To test this assay configuration, we screened a large (94,965 compounds), diverse (1,000 scaffolds that vary in representation from 20 to 100 compounds per chemotype), highly curated (PAINS, Lipinsky), lead-like (sp3-enriched, spirocyclic, novel chemotypes) chemical library (Genesis, NCATS) formatted as a 6 and 7-point interplate concentration response in 1536-well source plates. Screening was performed with the NCATS automation group using the fully-automated Kalypsis Robotic platform employing the same liquid dispensing, compound pinning, and endpoint measurement equipment as was used offline in the pilot screening. In this format, primary screening was performed on 409 plates over the course of 4 screening days. Screening day 2 included 200 plates translating to a peak throughput of over 40,000 compounds per day in 6 and 7-point concentration responses. The primary assay was robust (S/B = 17.6 ± 2.2, CV = 2.0% ± 0.6, and a Z′ of 0.93 ± 0.3; **Fig 14A**) as calculated from the control wells of each plate in the series.

**Fig 15:**
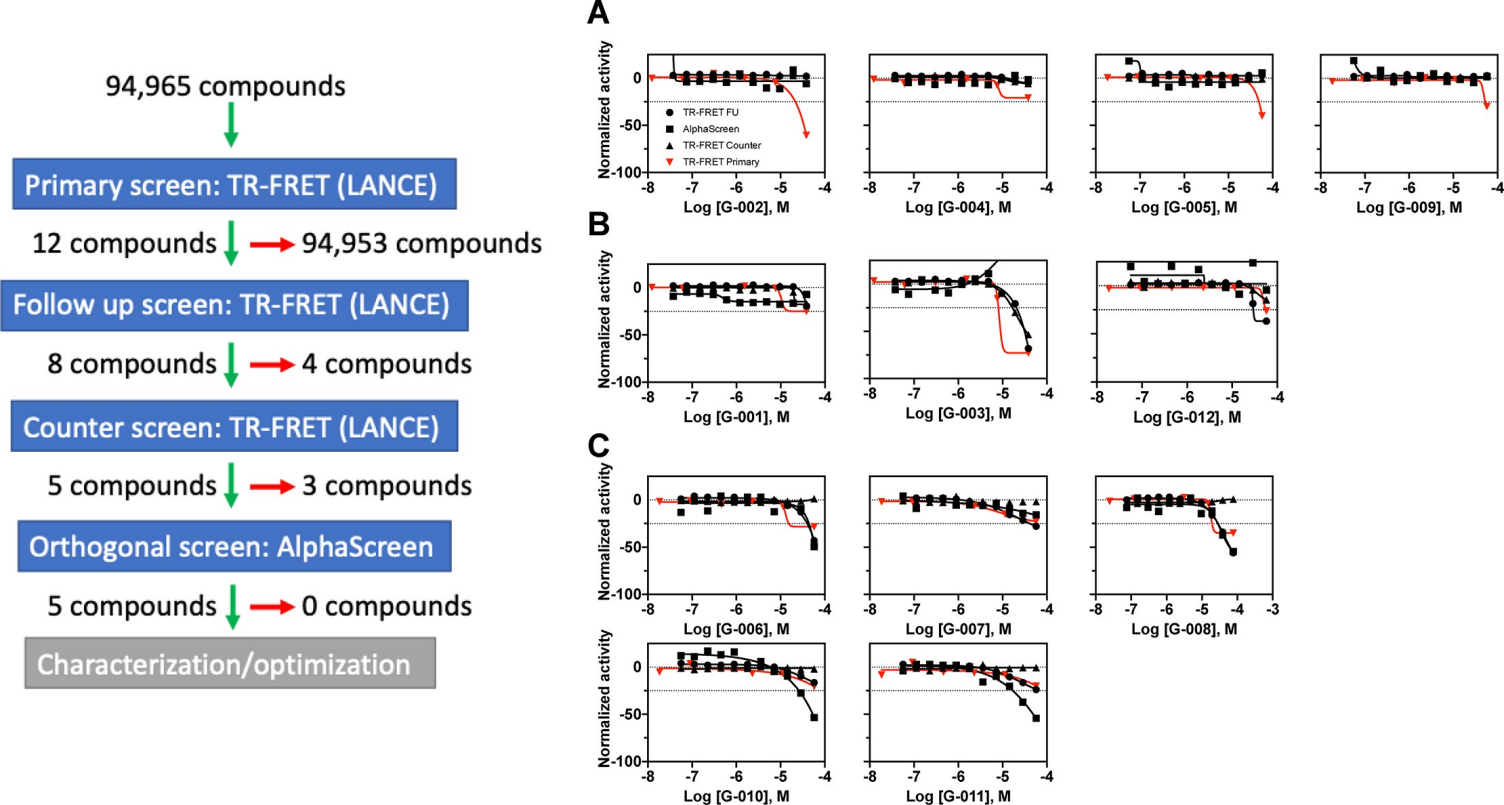
Large library screening workflow and results. Left panel: schematic of screening process showing input library size, assay utilization, advanced compounds (green arrow), and non-advanced compounds (red arrow). Right panel: activity profiles for compounds selected for follow-up testing from the primary screen. Activities are shown for each assay performed and the compound profiles are segregated by level of assay advancement. (A) No activity on TR-FRET follow up. (B) Unacceptable counter screen activity. (C) Confirmed active with AlphaScreen.

Due to the low level of activity in the screen, compounds were selected for follow-up studies if they exhibited a negative curve classification (all curves in **Fig 14B**) and a maximum normalized response of <-25% (red curves in **Fig 14B**). This resulted in 12 compounds selected for follow-up testing. These compounds, selected from chemical stocks, were used to make the library plates, plated in 11-point concentration response format ranging from 38 μM to 37 nM, and confirmed off-line using the TR-FRET primary screen assay (**Fig 15**). Offline testing confirmed 8 of the 12 actives identified in the primary screen. The 4 compounds that were not confirmed displayed curve class -4 and were active only at the highest concentration in the primary screen. These results provided another example of the value of qHTS format screening and the ability to use concentration-responsiveness as an early activity filter. The remaining active compounds were subjected to additional investigation using the TR-FRET-based counter screen assay to remove assay-interfering false positive compounds. Three compounds displayed unacceptable activity in the counter screen assay (same curve classification or maximum activity as the primary screen) and were removed from follow-up testing. The remaining compounds were then subjected to orthogonal assay testing using the AlphaScreen SIRPα-CD47 interaction assay and 5 were confirmed to have similar activity as in the primary screen. The chemical identities of the confirmed active compounds will be the subject of a follow-up report resulting from their ongoing characterization and optimization.

## Discussion

Herein, we describe the creation, validation, and successful piloting of a group of novel biochemical-based high-throughput screening assays capable of surveying large chemical libraries for molecules that modulate the SIRPα-CD47 interaction. Whereas several biologics (antibodies and decoy receptors) have been generated against CD47 and SIRPα, small molecule inhibitors of their interaction have yet to be described. Despite the advancement of several biologic-based CD47 inhibitors into clinical testing (Phase 1 and 2), small molecules have the potential to offer critical advantages in terms of ligand specificity (SIRPα vs. TSP1 blockade), pharmacokinetic flexibility, tissue distribution, regulatory complexity, manufacturability, and cost. To enable the discovery, characterization and development of novel SIRPα-CD47 inhibiting small molecules, we sought to design a high-throughput screening platform capable of screening diverse small molecule libraries by first evaluating several different biochemical screening technologies coupled with recombinant SIRPα and CD47 constructs.

Our goal was to screen large chemical libraries (>100,000 molecules) in a concentration response format (qHTS), using 1536 well plates, and in a fully automated manner. Our choice of assay technologies reflects these parameters along with their history of use in conducting protein-protein interaction assays [67,103]. All three assay technologies were optimized to robust performance as evident in Z’ factor and pilot screening performance. However, the ability to detect active compounds in library screening is a balance of statistical performance (Z’ factor) and assay sensitivity (compound activity rate) [104]. In this case, the optimized assays achieved robust statistical performance (Z’ > 0.8) but displayed varying activity rates in LOPAC pilot screening (0.2% for LANCE TR-FRET and 1.1% for CisBio TR-FRET and AlphaScreen). Of these actives, none were confirmed as hits after counter and orthogonal testing. This result led us to hypothesize that the increased activity rates seen in the CisBio TR-FRET and AlphaScreen assays would lead to a greater accumulation of false positives in larger library primary screening. Accordingly, we chose the LANCE TR-FRET assay for primary screening as it offered the best balance of statistical robustness and low false positive rate. However, as pointed out in Inglese et al. [104], these characteristics are likely to lead to a low sensitivity screening assay. As a predictor of sensitivity, the ratio of the observed positive control compound potency vs. the expected potency (IC50’/IC50) of greater than 3-fold coupled with a high Z’ factor (>0.5) is likely to lead to an insensitive screening assay mostly as a result of increased avidity of the binding partners being presented as multivalent reagents. In our case, we can infer an IC50’/IC50 of 15, 5, and 2.8 for the CisBio, LANCE, and AlphaScreen assays respectively using the SIRPα-CD47 *K*_*d*_ in **Fig 2B** as *IC50* and the activity of “SIRPα-cold” in **Fig 5**. As the Z’ factor for all assays was well above 0.5, predicted sensitivity would be borderline (AlphaScreen) to low (CisBio and LANCE) for our assays. This perhaps underlies the low activity rate observed in large drug-like library screening effort using the LANCE TR-FRET assay. In this study, the reduction of false positive activity was prioritized over total activity rate.

**Fig 16:**
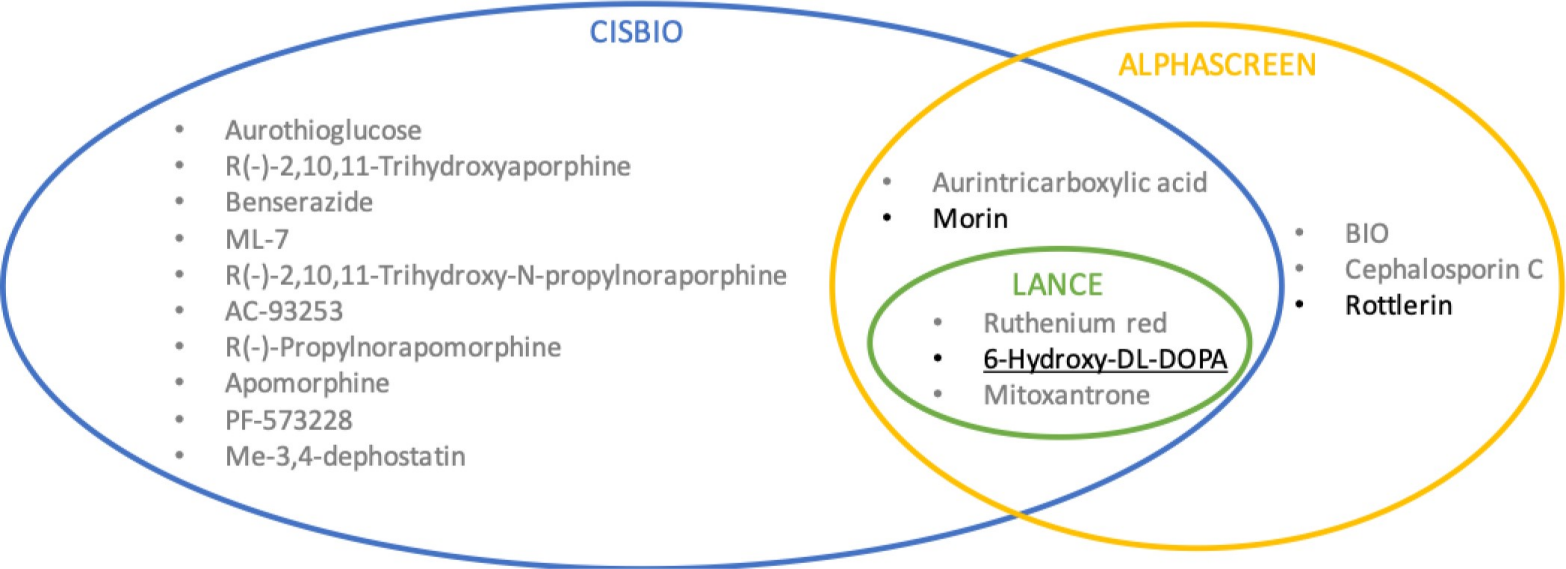
Venn diagram of LOPAC active compounds in all screening assays. Grey denotes compounds triaged in counter screening. Black denotes remaining active compounds.

Another factor influencing the assay design and screening platform was the capability to efficiently identify assay-interfering compounds that are ubiquitous in large chemical libraries. Thus, to limit what is to be screened, we placed less emphasis on initial cheminformatic filtering and with it, potential filter bias and false negatives. Instead, we built a complementary set of primary and counter screening assays using data analyses to objectively exclude assay-interfering molecules from experimental actives. Many recent commentaries point out the overly simplistic and potentially counterproductive use of PAINS filters to triage libraries prior to screening [105–108]. As mentioned before [108], it is important to have a strategy in place that can differentiate between compounds that are interfering with the assay technology (Type 1) or with the target via an undesirable mechanism (Type 2). In our case, the LANCE TR-FRET assay provided the most logical path to follow-up studies. As was previously pointed out, the ability to use high screening compound concentrations is an advantage of TR-FRET-based assays where compound interference is reduced by the lanthanide fluorescence lifetime and FRET read delay. This allows screening at high compound concentrations to reduce false negative occurrences without increasing false positives [109]. Furthermore, in pilot and large library screening we integrated the LANCE TR-FRET primary screening assay together with the AlphaScreen assay as a follow-up orthogonal assay to catch Type 1 false positives, as well as counter screening assays in both the TR-FRET assay and AlphaScreen to catch Type 2 false positives. The decision to favor the LANCE assay over the CisBio assay is based on sensitivity versus false positive rate. While the CisBio assay appeared to be more sensitive (15 actives versus 3 for LANCE), only 1 of 15 actives was advanced for follow-up studies versus 1 out of 3 for LANCE. Based on this rate, the LANCE assay appeared less susceptible to assay interference during LOPAC screening. The decision to favor the LANCE technology over the AlphaScreen in primary screening was due to the more efficient advancement of compounds to follow-up as well as cost. To triage actives in the AlphaScreen, a second counter-screening assay was required to remove interfering compounds, thus increasing time and costs. One caveat to this is that the unique active compounds that were elucidated in the AlphaScreen and not the TR-FRET assays (**Fig 16**) could be a more accurate set of actives. However, these were triaged by other undesirable characteristics such as Hill slope and reactivity/promiscuity. The AlphaScreen assay, as used in our format, has additional liabilities compared to the TR-FRET assays. These include the increased susceptibility to interference of the Ni^2+^-NTA to His-tag binding versus the anti-6His antibody on the TR-FRET assays, and the susceptibility to singlet oxygen quenching. Regardless, we would expect real hits derived from the TR-FRET assay to also be active in the AlphaScreen assay making it an excellent candidate for an orthogonal confirmatory assay.

The value of combining these assays with a qHTS-formatted large library screen is evident in the example described herein. Multi-day fully-automated primary screening was robust and highly discriminatory and active compounds were efficiently triaged using confirmatory, counter screen, and orthogonal testing. Interestingly, all 5 confirmed compounds with similar potency displayed a greater maximum activity in the AlphaScreen assay than in the TR-FRET assay. This could point to a greater sensitivity of the SIRPα-CD47 PPI in the AlphaScreen assay than the TR-FRET assay perhaps due to the vast differences in size and diffusion of the protein-bound beads to the compounds—something evident in the validation testing using SIRPα-cold and biotin as inhibitors. *In vivo*, the SIRPα-CD47 interaction occurs between two adjacent cells, perhaps making the AlphaScreen assay a better model of their biological interaction than the lower valency TR-FRET assays.

Our primary screen activity rate was very low (0.01%) but consistent with the challenges of targeting PPIs with traditional lead-like chemical libraries and assays with inherently low sensitivity due to multivalent reagents. However, it is important to point out the maximum capacity of these assays for efficient follow-up testing. Using an 11-point concentration response format, each 1536-well plate can accommodate 128 distinct compounds in intra-plate format. In a one-person day (8 hours) of offline testing, we tested up to 14 plates in each of the described assays. This translates to a throughput of at least 1792 compounds tested (formatted with intraplate controls) per day as follow-up or the ability to screen a 100,000-compound library and fully triage (confirm, counter, orthogonal) active compounds at a 2% hit rate in 7 testing days. This also critically depends on efficient and effective informatics and compound management support as is the case at the NCATS screening center. Using the assays as described supports the screening of larger and more diverse libraries with greater potential to result in successful PPI modulator identification. It will also permit the identification of more active compounds with relaxed chemical or activity criteria to more objectively assess their specificity and *lower false-negative rates* in unbiased/rapid/robust follow-up testing. For instance, increasing the number of compounds undergoing counter screening will provide valuable hard data to cross reference with predictions of assay interference activities to bolster efforts as discussed above [108]. Concerning the low activity rate of screening typical lead-like chemical libraries for PPI modulators as exemplified herein, these assays could also serve as indispensable tools in the characterization and development of more potent active compounds obtained from screening libraries of greater molecular complexity like macrocycles or peptoids [110] or more ligand efficient lead-like compounds derived from fragment screening campaigns [111].

In summary, we have developed and validated biochemical TR-FRET- and AlphaScreen-based assays for screening small molecule inhibitors of SIRPα-CD47 interaction. To our knowledge, these are the first HTS assays to target the SIRPα-CD47 PPI. These assays will be integrated into a platform capable of screening large drug-like chemical libraries to discover pre-therapeutic molecules as well as rank order lead candidates following medical chemistry optimization. Likewise, the process outlined herein from protein reagent creation to library screening is a viable approach to discover small molecule modulators for other emerging PPI targets.

## Supporting information

S1 File SUPPLEMENTAL INFORMATION. Materials and methods describing the SAXS studies.
(DOCX)

S1 Fig SIRPα and CD47 recombinant protein production. (A) Representative SDS PAGE showing SIRPα purity following biotin ligation and final purification using ion exchange chromatography (QHP). Upper band in lane marked “Post Dial#2” is the biotin ligase BirA (B*). Lower bands ($) are SIRPα-biotin. (B) Representative SDS PAGE showing CD47-CD4-6His production prior to final purification using size exclusion chromatography. Lanes labeled “M” in (A) and (B) contain molecular mass markers with sizes indicated in kDa. (C) Table describing protein reagent production.
(TIF)

S2 Fig Mass spectrometry showing biotin incorporation into SIRPα. (A) HPLC-MS retention time tracings for Total Ion Chromatogram and 280 nm absorbance. (B) Positive Ion scan showing mass to charge ratio (m/z) of species present in the peak at 3.540–3.739 min. (C) abundance of deconvoluted masses present in the peak at 3.540–3.739 min. Note SIRPα without biotin has a mass of 15894 Da and with biotin has a mass of 16120 Da.
(TIF)

S3 Fig SAXS analysis of SIRPα-Avi. (A) Experimental SAXS data for 3 mg/mL (orange) and 4 mg/mL (blue) SIRPα-Avi samples. (B,C) Guinier plots for 3 mg/mL (orange) and 4 mg/mL (blue) SIRPα-Avi samples. (D) Dimensionless Kratky plots show a slight peak shift for SIRPα-Avi. (E) Pair distribution function (P(r)) calculated from SAXS profiles in (A). (F) Fit between experimental data and fitted data using SCÅTTER. (G) Fit and error-weighted residuals of experimental (black dots) and theoretical SAXS profile for the modeled SIRPα-Avi (red) performed with FOXS. (H) Superimposition of the modeled SIRPα-Avi structure (cartoon) and the averaged SAXS reconstruction with DAMMIN (surface). The loops involved in the interaction with CD47 are colored in orange and labeled according to their residue numbers. The N- and C-terminal residues are labeled.
(TIF)

S4 Fig CisBio TR-FRET assay optimization. (A) Titration of donor and acceptor reagents. (B) Comparison of plate type. (C) Signal stability over time.
(TIF)

S5 Fig LANCE TR-FRET optimization. (A) Titration of acceptor and donor reagents. (B) Positive control inhibitor (SIRPα-cold) IC50 titration at different donor:acceptor ratios. (C) Acceptor titration at optimal 1X donor level. (D) Positive control inhibitor (SIRPα-cold) IC50 titration at different acceptor levels as in (C). (D) Table of donor and acceptor molar concentrations.
(TIF)

S6 Fig LANCE TR-FRET assay order of addition and stability studies. (A) Assay performance based on order of reagent addition, acceptor then donor (A+D) or donor then acceptor (D+A). (B) Assay signal stability at 0 and 48 h. (C) Stability of positive control inhibitor potency at 0 and 48 h.
(TIF)
