# Supplementary material for: Quantitative high-throughput screening assays for the discovery and development of SIRPα-CD47 interaction inhibitors
Source: PLoS One. 2019 Jul 5;14(7):e0218897. doi: 10.1371/journal.pone.0218897 (PMC6611588; doi:10.1371/journal.pone.0218897)
Supplement: S1 File — Materials and methods describing the SAXS studies. (DOCX) [file pone.0218897.s001.docx]

**SUPPLEMENTAL INFORMATION**

**MATERIALS AND METHODS**

SAXS data were collected at the Advanced Light Source 12.3.1 beamline (SIBYLS). The energy was 11 keV and the sample-to-detector distance was 1.5 m, resulting in scattering vectors, *q*, ranging from 0.01 to 0.5 Å_-1_ (*q* is defined as 4πsinθ, where 2θ is the scattering angle). All experiments were performed on 20 μL samples at 20 °C using a Hamilton robot for loading samples from the 96-well plate. SIRPα-Avi was purified by size-exclusion chromatography before SAXS experiments using a 24-ml S75 column (GE Healthcare). SAXS data were collected on concentrated fractions (ranging from 2 to 4 mg/ml). Buffer used for size-exclusion chromatography was used for buffer subtraction. For each sample, continuous snapshots of 0.3sec/frame for 10 sec (total of 30 frames) were taken, and data were monitored for radiation damage. We checked the data accuracy by comparing radius of gyration *Rg* obtained by Guinier approximation (I(q)=I(0)exp(-q^2^Rg^2^/3) with *qRg*<1.6 for each sample concentration. At all concentrations, we obtained *Rg* values that deviate by less than 0.05 Å. (19.4 ±0.194, 19.43 ±0.195, and 19.3 ± 0.119), and Guinier plots that are linear, indicating negligible interference from structure factor or aggregation. The 2 mg/mL data was too noisy and not processed further. Data were analyzed with SCATTER (LBNL BL12.3.1 software) and GNOM (Svergun, 1992) to generate Guinier, Kratky, and P(r) plots and determine *Rg* (radius of gyration), *I*(0), and *D*max (maximum dimension of the particle). The parameters obtained for SIRPα-avi at both concentrations are shown in **S1 Table**.

**S1 Table**. Dimensions parameters for SIRPα-Avi obtained by SAXS

| Conc (mg/mL) | Rg(rec) | Rg(real) | I(0) (rec) | I(0) real | Dmax |
| --- | --- | --- | --- | --- | --- |
| 3 | 18.25 Å | 21.26 Å | 3510 | 3642 | 58 Å |
| 4 | 18.05 Å | 20.73 Å | 4350 | 4660 | 58 Å |

Analysis of the dimensionless Kratky plots (**S3D Fig**) reveals a maximum that is slightly shifted from the ideal value of 1.1 at qR_g_=√3, indicating a folded protein with a flexible tail (Durand, 2010). We generated 10 ab initio 3D envelopes with the program DAMMIN (Svergun, 1999) and averaged them with the program DAMAVER. The 10 reconstructions deviated little from each other (NSD=0.396; standard deviation of NSD=0.075), indicating robustness of the reconstruction over several independent runs. We used the x-ray structure of SIRPα (PDBcode 2WNG, residues 1-118) as a starting model to build the missing C-terminal Avi-tag residues with SWISSMODEL (Waterhouse, 2018). The results from the modeling step suggest that the C-terminal tag adopts a partially folded conformation. The experimental SAXS data and the calculated SAXS curve obtained from the modeled SIRPα-Avi construct were compared with FOXS (Schneidman-Duhovny, 2016). We determined an overall χ^2^ parameter of 0.27, which reflects a good fit of the experimental curve to the calculated curve (**S3F Fig**). In addition, the superimposition of the averaged reconstructed envelope with the model generated with SWISSMODEL for SIRPα-Avi shows a good agreement between the model and the SAXS envelope (**S3H Fig**). It further reveals that the protein is folded with a flexible tail, which corresponds to the C-terminal Avi-tag located far from the regions that are known to interact with CD47. These results confirm that this construct is well suited for our binding assays.

**REFERENCES**:

Svergun, D.I., 1999. Restoring low resolution structure of biological macromolecules from solution scattering using simulated annealing. Biophys J 76, 2879–2886.

Svergun, D.I., 1992. Determination of the regularization parameter in indirect-transform methods using perceptual criteria. J. Appl. Crystallogr. 25, 495–503. https://doi.org/10.1107/s0021889892001663

Durand D, Vivès C, Cannella D, Pérez J, Pebay-Peyroula E, Vachette P, Fieschi F. NADPH oxidase activator p67(phox) behaves in solution as a multidomain protein with semi-flexible linkers. J Struct Biol. 2010 Jan; 169(1):45-53.)

Waterhouse, A., Bertoni, M., Bienert, S., Studer, G., Tauriello, G., Gumienny, R., Heer, F.T., de Beer, T.A.P., Rempfer, C., Bordoli, L., Lepore, R., Schwede, T. SWISS-MODEL: homology modelling of protein structures and complexes. Nucleic Acids Res. 46(W1), W296-W303 (2018).

Schneidman-Duhovny D., Hammel M., Tainer J.A., Sali A.FoXS, FoXSDock and MultiFoXS: single-state and multi-state structural modeling of proteins and their complexes based on SAXS profiles. Nucleic Acids Res. 2016; 44: W424-W429.
