# Supplementary figures and images for: Quantitative high-throughput screening assays for the discovery and development of SIRPα-CD47 interaction inhibitors
Source: PLoS One. 2019 Jul 5;14(7):e0218897. doi: 10.1371/journal.pone.0218897 (PMC6611588; doi:10.1371/journal.pone.0218897)

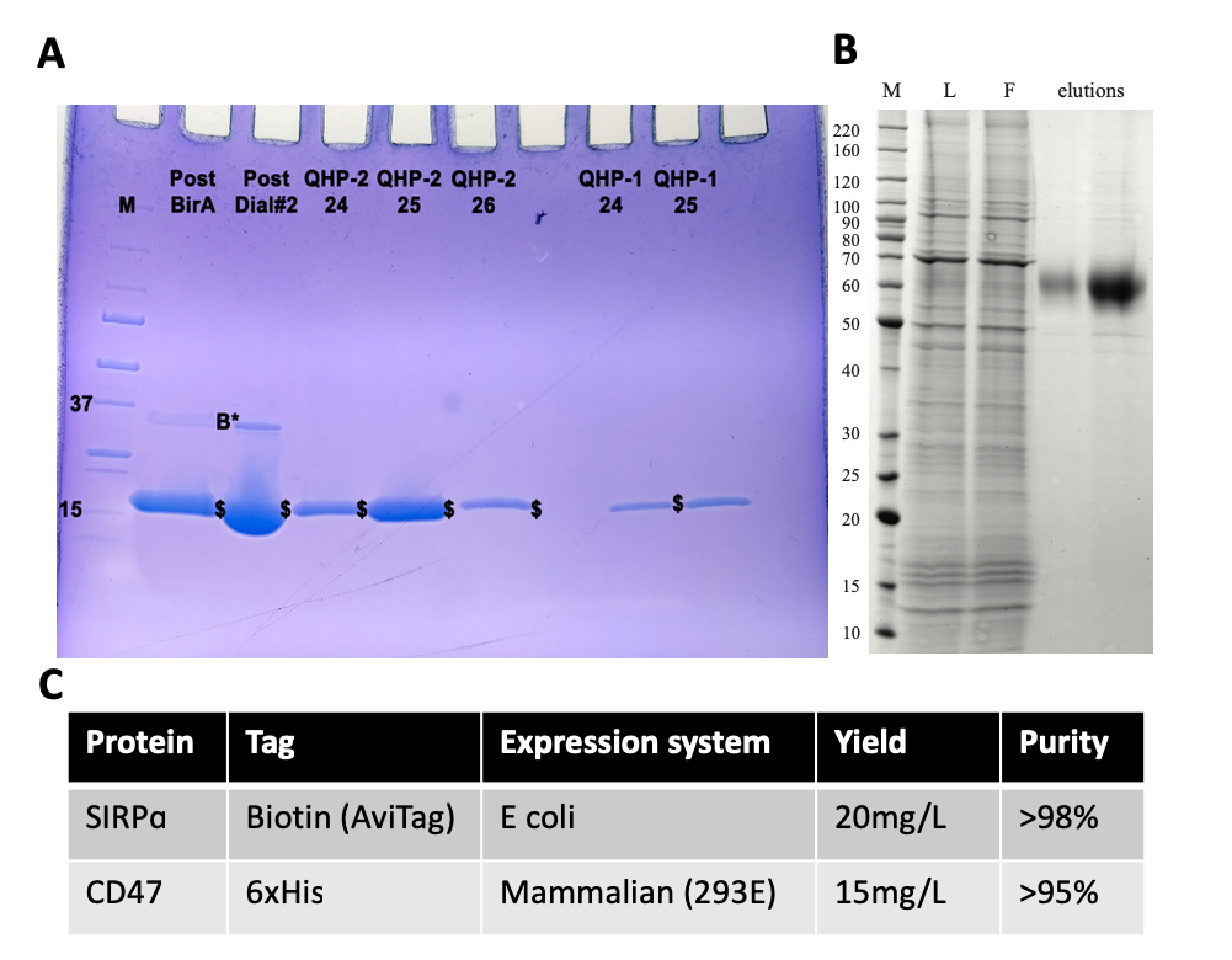

Supplement: S1 Fig — (A) Representative SDS PAGE showing SIRPα purity following biotin ligation and final purification using ion exchange chromatography (QHP). Upper band in lane marked “Post Dial#2” is the biotin ligase BirA (B*). Lower bands ($) are SIRPα-biotin. (B) Representative SDS PAGE showing CD47-CD4-6His production prior to final purification using size exclusion chromatography. Lanes labeled “M” in (A) and (B) contain molecular mass markers with sizes indicated in kDa. (C) Table describing protein reagent production. (TIF) [file pone.0218897.s002.tif]

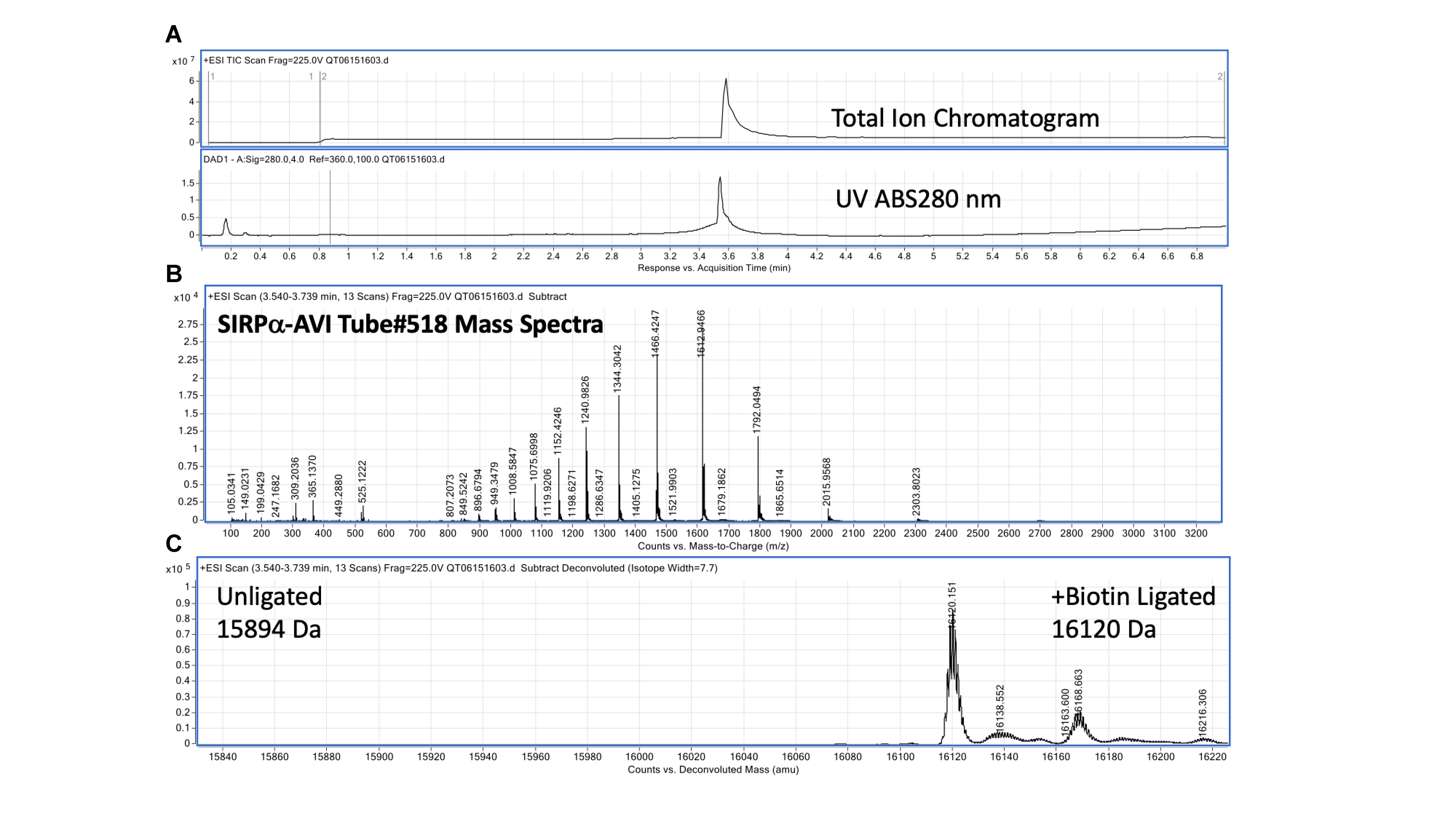

Supplement: S2 Fig — (A) HPLC-MS retention time tracings for Total Ion Chromatogram and 280 nm absorbance. (B) Positive Ion scan showing mass to charge ratio (m/z) of species present in the peak at 3.540–3.739 min. (C) abundance of deconvoluted masses present in the peak at 3.540–3.739 min. Note SIRPα without biotin has a mass of 15894 Da and with biotin has a mass of 16120 Da. (TIF) [file pone.0218897.s003.tif]

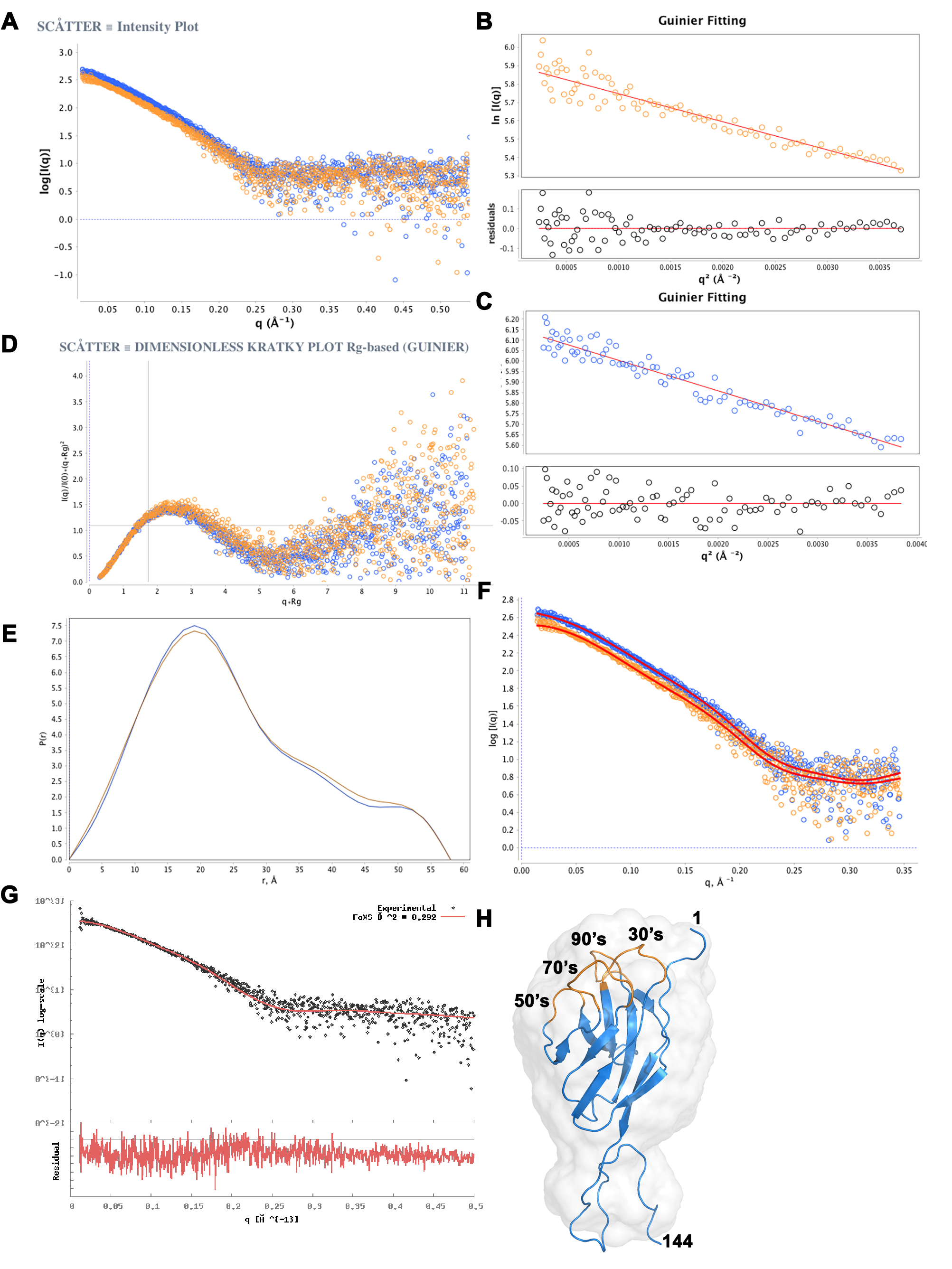

Supplement: S3 Fig — (A) Experimental SAXS data for 3 mg/mL (orange) and 4 mg/mL (blue) SIRPα-Avi samples. (B,C) Guinier plots for 3 mg/mL (orange) and 4 mg/mL (blue) SIRPα-Avi samples. (D) Dimensionless Kratky plots show a slight peak shift for SIRPα-Avi. (E) Pair distribution function (P(r)) calculated from SAXS profiles in (A). (F) Fit between experimental data and fitted data using SCÅTTER. (G) Fit and error-weighted residuals of experimental (black dots) and theoretical SAXS profile for the modeled SIRPα-Avi (red) performed with FOXS. (H) Superimposition of the modeled SIRPα-Avi structure (cartoon) and the averaged SAXS reconstruction with DAMMIN (surface). The loops involved in the interaction with CD47 are colored in orange and labeled according to their residue numbers. The N- and C-terminal residues are labeled. (TIF) [file pone.0218897.s004.tif]

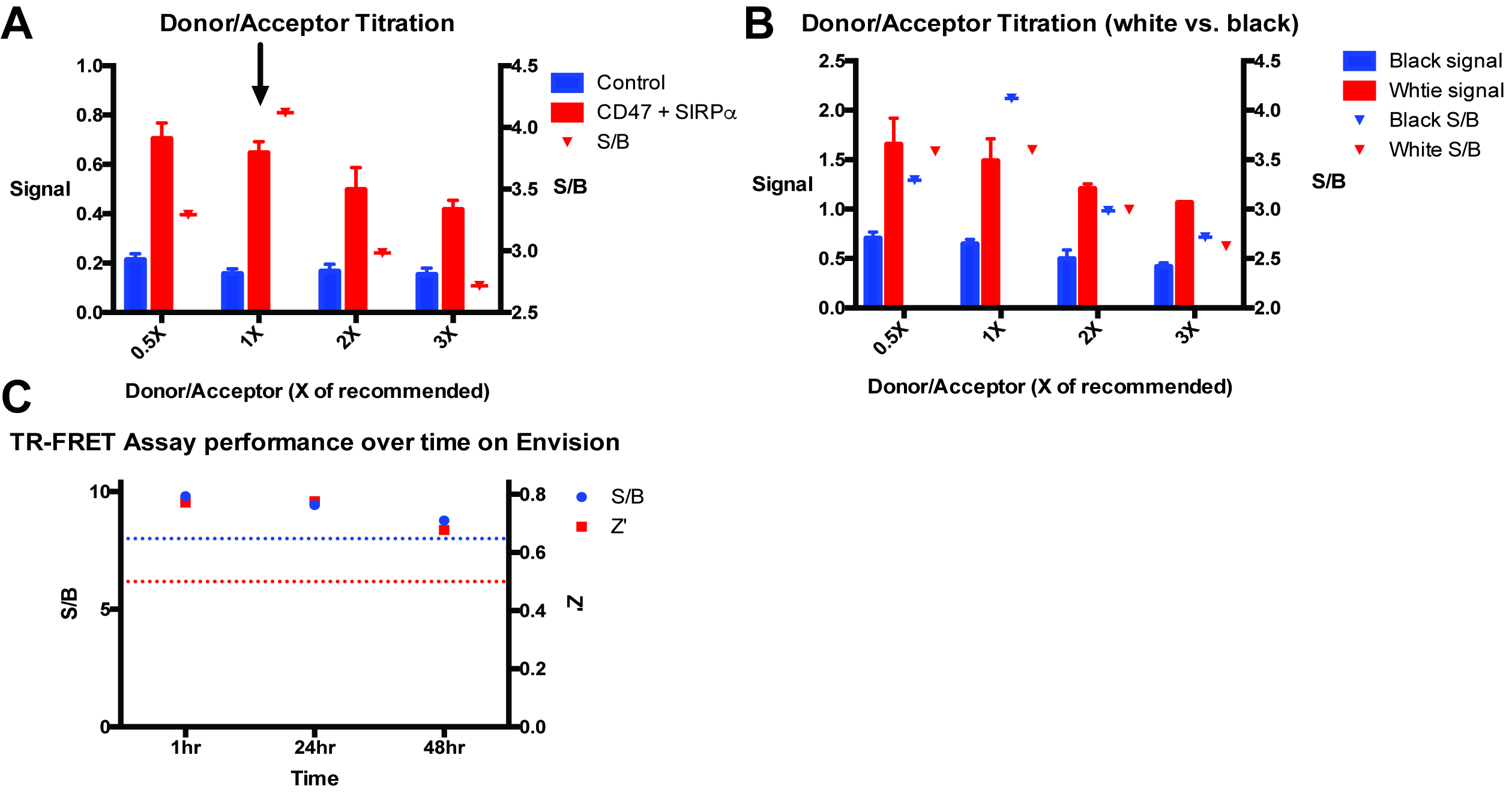

Supplement: S4 Fig — (A) Titration of donor and acceptor reagents. (B) Comparison of plate type. (C) Signal stability over time. (TIF) [file pone.0218897.s005.tif]

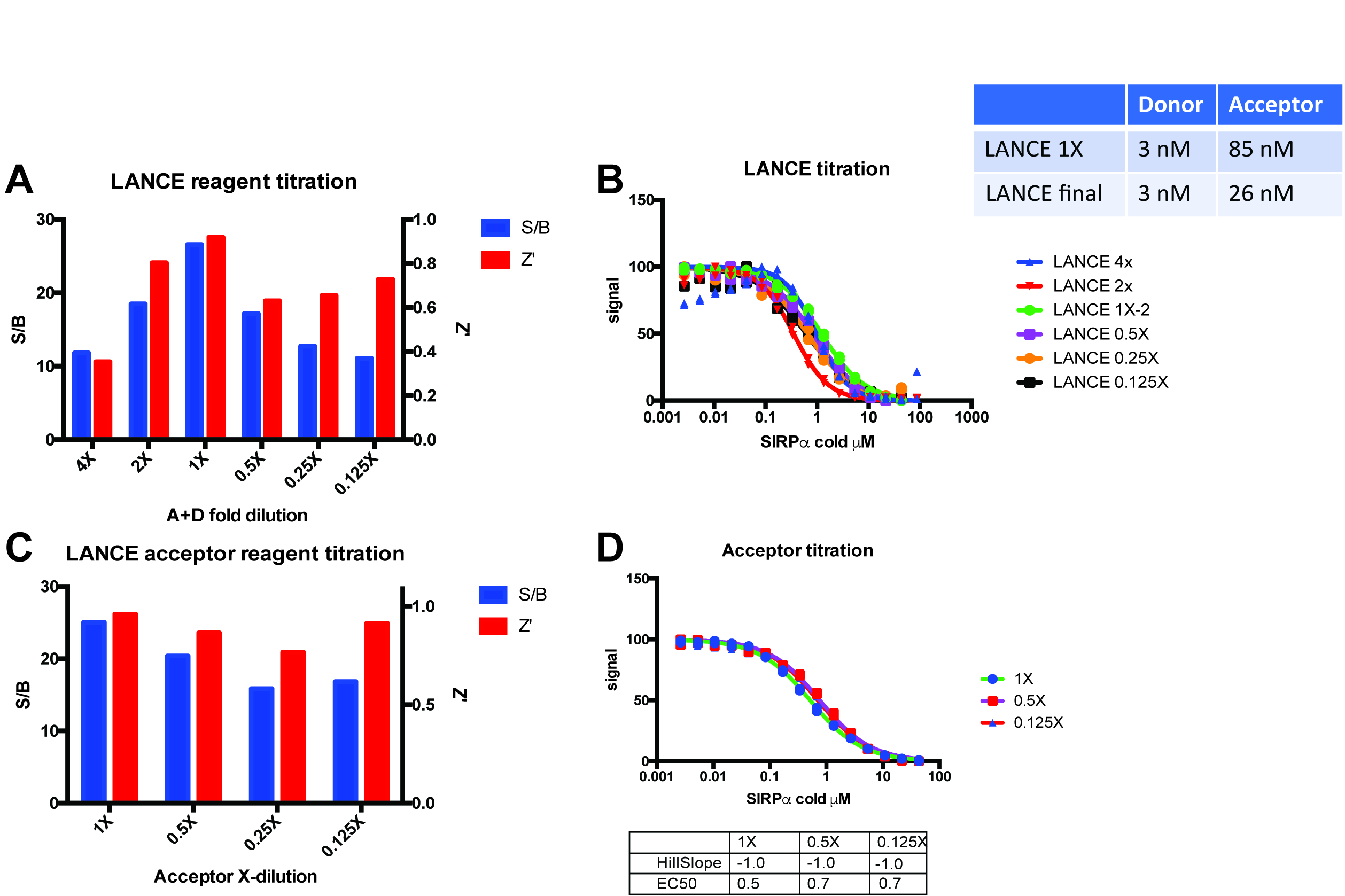

Supplement: S5 Fig — (A) Titration of acceptor and donor reagents. (B) Positive control inhibitor (SIRPα-cold) IC50 titration at different donor:acceptor ratios. (C) Acceptor titration at optimal 1X donor level. (D) Positive control inhibitor (SIRPα-cold) IC50 titration at different acceptor levels as in (C). (D) Table of donor and acceptor molar concentrations. (TIF) [file pone.0218897.s006.tif]

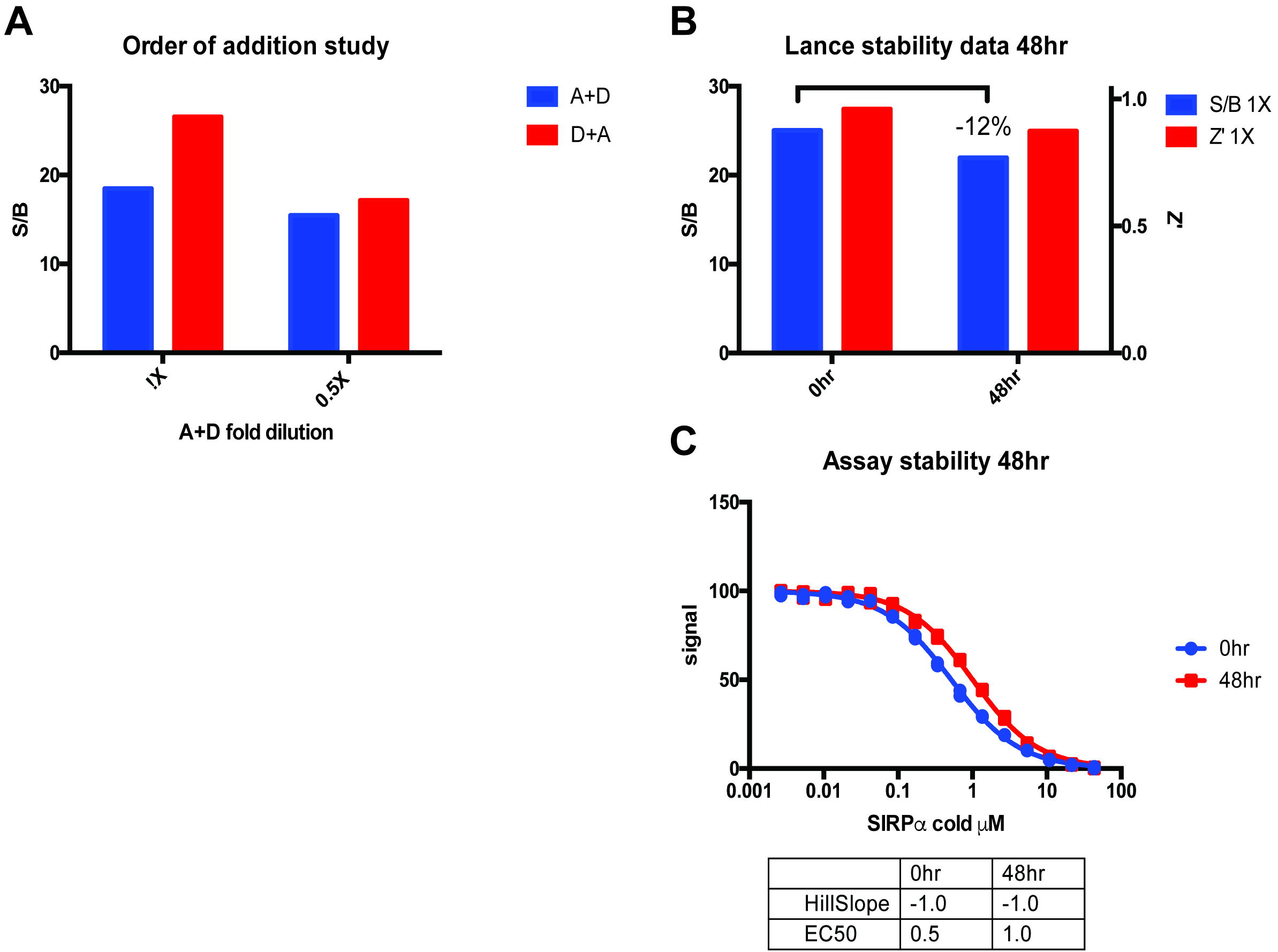

Supplement: S6 Fig — (A) Assay performance based on order of reagent addition, acceptor then donor (A+D) or donor then acceptor (D+A). (B) Assay signal stability at 0 and 48 h. (C) Stability of positive control inhibitor potency at 0 and 48 h. (TIF) [file pone.0218897.s007.tif]
